# Direct Competition between hnRNP C and U2AF65 Protects the Transcriptome from the Exonization of *Alu* Elements

**DOI:** 10.1016/j.cell.2012.12.023

**Published:** 2013-01-31

**Authors:** Kathi Zarnack, Julian König, Mojca Tajnik, Iñigo Martincorena, Sebastian Eustermann, Isabelle Stévant, Alejandro Reyes, Simon Anders, Nicholas M. Luscombe, Jernej Ule

**Affiliations:** 1 European Molecular Biology Laboratory (EMBL) European Bioinformatics Institute, Wellcome Trust Genome Campus, Hinxton, Cambridge CB10 1SD, UK; 2 MRC Laboratory of Molecular Biology, Hills Road, Cambridge CB2 0QH, UK; 3 Faculty of Medicine, University of Ljubljana, Vrazov trg 2, SI-1104 Ljubljana, Slovenia; 4 EMBL, Genome Biology Unit, Meyerhofstraße 1, 69117 Heidelberg, Germany; 5 UCL Genetics Institute, Department of Genetics, Environment and Evolution, University College London, Gower Street, London WC1E 6BT, UK; 6 Cancer Research UK London Research Institute, 44 Lincoln’s Inn Fields, London WC2A 3LY, UK; 7 Okinawa Institute for Science and Technology Graduate University, 1919-1 Tancha, Onna-son, Kunigami-gun, Okinawa 904-0495, Japan

## Abstract

There are ∼650,000 *Alu* elements in transcribed regions of the human genome. These elements contain cryptic splice sites, so they are in constant danger of aberrant incorporation into mature transcripts. Despite posing a major threat to transcriptome integrity, little is known about the molecular mechanisms preventing their inclusion. Here, we present a mechanism for protecting the human transcriptome from the aberrant exonization of transposable elements. Quantitative iCLIP data show that the RNA-binding protein hnRNP C competes with the splicing factor U2AF65 at many genuine and cryptic splice sites. Loss of hnRNP C leads to formation of previously suppressed *Alu* exons, which severely disrupt transcript function. Minigene experiments explain disease-associated mutations in *Alu* elements that hamper hnRNP C binding. Thus, by preventing U2AF65 binding to *Alu* elements, hnRNP C plays a critical role as a genome-wide sentinel protecting the transcriptome. The findings have important implications for human evolution and disease.

## Graphical abstract

**Graphical Abstract:**

**Figure:**
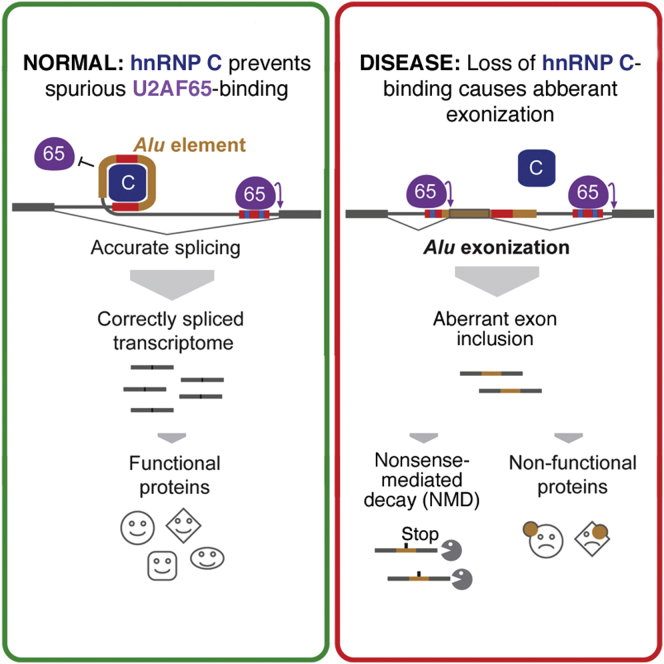

**Highlights:**

► Quantitative iCLIP reveals genome-wide competition of hnRNP C and U2AF65 ► hnRNP C is a global repressor of aberrant exonization of thousands of *Alu* elements ► Disease-associated mutations in *Alu* elements hinder hnRNP C-dependent repression ► Selection reinforces strong hnRNP C binding to contain *Alu* exonization

## Teaser

The RNA-binding protein hnRNP C prevents the formation of aberrant *Alu* exons by blocking the binding of the splicing factor U2AF65 to potential *Alu* splice sites. Breakdown of this system leads to expression of thousands of harmful exons and to human disease.

## Introduction

Most eukaryotic primary transcripts consist of short exons and very long introns. This exon-intron structure provides important opportunities for proteome diversity and evolution. The selective usage of exons through alternative splicing is a major source of proteome diversity in higher organisms (Nilsen and Graveley, 2010). Furthermore, the long intronic regions facilitate the creation of new protein functionalities through exon shuffling by nonallelic recombination between different genes (Fedorova and Fedorov, 2003; Xing and Lee, 2006).

The complex gene structure means that nascent transcripts must be carefully processed before they can be used. In particular, the precise removal of introns in the splicing reaction demands the complex interplay between a multitude of *trans*-acting factors and *cis*-regulatory splicing signals in the premessenger RNA (pre-mRNA) molecule. Among the former, a multisubunit complex called the spliceosome catalyzes intron excision and exon joining at splice sites (Wahl et al., 2009). Among the latter, the GU and AG dinucleotides, flanked by additional sequence elements, define intron-exon boundaries at the 5′ and 3′ splice sites. The most prominent flanking element is a sequence rich in uridines and cytidines, known as the polypyrimidine tract, which is located immediately upstream of the 3′ splice site, and is required for binding of the U2 auxiliary factor 65 (U2AF65). Binding of U2AF65 is essential for recruiting the small nuclear ribonucleoprotein (snRNP) U2, a component of the spliceosome, and thus comprises a major regulatory event during 3′ splice-site recognition (Wahl et al., 2009).

Although the splicing reaction is carried out with very high precision, the *cis*-acting signals that mediate spliceosome binding show limited sequence constraint. As a result, pre-mRNAs harbor a large number of potential splice sites with very similar sequences to true splice sites, but that are not used under normal conditions. Importantly, these cryptic splice sites can act as a source of new exons during evolution. On the other hand, uncontrolled recognition of cryptic splice sites can have deleterious consequences for the cell if it creates aberrant transcripts, and the inclusion of cryptic exons has been implicated in various diseases (Buratti et al., 2007; Dhir and Buratti, 2010; Vorechovský, 2006). It is therefore imperative for the cell to tightly control the accessibility of such signals to the splicing machinery.

Candidates for masking cryptic splice sites are the heterogeneous nuclear ribonucleoproteins C1/C2 (referred to as hnRNP C). hnRNP C is abundant in the nucleus and associates with all nascent transcripts (Beyer et al., 1977; König et al., 2010). It forms hnRNP particles, which have been described to compact large regions of pre-mRNA and have been implicated in the regulation of alternative splicing (Choi et al., 1986; Dreyfuss et al., 1993). In order to investigate the function of hnRNP C on a genomic scale, we previously developed a technique called individual-nucleotide resolution UV-crosslinking and immunoprecipitation (iCLIP) (König et al., 2010, 2011). Using iCLIP, we characterized the transcriptome-wide binding pattern of hnRNP C at an unprecedented resolution and discovered that hnRNP C represses alternative exons by binding next to the splice sites (König et al., 2010). However, the mechanism by which this repression is achieved, and its importance in repressing cryptic exons, remained unclear.

Here, we introduce iCLIP as a high-resolution, quantitative technique that enables us to measure how the competition between hnRNP C and the core splicing factor U2AF65 regulates the inclusion of alternative exons on a genomic scale. Notably, we show that hnRNP C blocks U2AF65 from cryptic 3′ splice sites, thereby preventing the aberrant expression of cryptic exons. Finally, we dissect how the differences in sequence specificities of hnRNP C and U2AF65 enable the splicing machinery to discriminate cryptic splice sites from genuine exons, and reveal the importance of hnRNP C for maintaining transcriptome integrity and preventing disease.

## Results

### hnRNP C and U2AF65 Bind at 3′ Splice Sites

**Figure S1:**
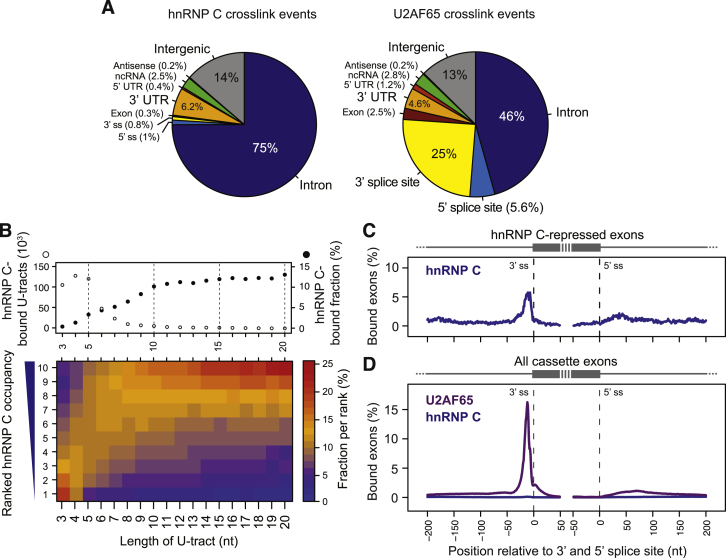
iCLIP Data Show Binding of hnRNP C and U2AF65 at 3′ Splice Sites of Target Exons, Related to Figure 1 (A) The majority of hnRNP C and U2AF65 crosslink events is located within introns. Pie chart summarizing the fraction of crosslink events within different genomic regions. 3‘ splice site (3′ ss) and 5‘ splice site (5′ ss) indicate the regions 200 nt upstream and downstream of Ensembl-annotated exons, respectively. (B) Binding sites on longer U-tracts show higher hnRNP C occupancy. Top panel: absolute numbers of hnRNP C-bound U-tracts of different length in the transcriptome (open circles) and the corresponding fraction of bound tracts (filled circles). Lower panel: heatmap showing the fraction of U-tracts of a given length that is allocated to the ten different ranks of hnRNP C occupancy. 3-nt U-tracts show the highest enrichment at the 10% weakest hnRNP-C-binding sites (rank 1), whereas U-tracts of nine or more nucleotides show the highest enrichment at the 10% strongest binding sites (rank 10). (C) hnRNP C binding is enriched immediately upstream of the 3′ splice sites of hnRNP-C-repressed exons. RNA map showing the percentage of exons that have a crosslink nucleotide at a certain position relative to the 3′ and 5′ splice site of all exons that are repressed by hnRNP C (taken from our RNA-seq data, Table S2). (D) U2AF65 binding is strongly enriched immediately upstream of the 3′ splice sites of all annotated exons. RNA map as in (C) visualizing U2AF65 and hnRNP C iCLIP data relative to all 3′ and 5′ splice sites annotated in the Ensembl database.

To investigate the detailed molecular function of hnRNP C, we first explored the potential for iCLIP to provide quantitative measurements of protein-RNA interactions. With an optimized protocol, we identified a total of 14 million unique hnRNP C crosslink events in untreated HeLa cells, which cluster into 438,360 binding sites (Table S1 and Figure S1A available online). This represents a 22-fold increase in crosslink events compared with our previously published data (König et al., 2010). The greatly increased complexity of the new data set allowed us to rank binding sites by their normalized occupancy, and to estimate the strength of hnRNP C-RNA associations. We find that the strongest binding sites reside at continuous uridine tracts (U-tracts) of nine or more uridines (Figure S1B, bottom). Overall, hnRNP C binds to more than 10% of all U-tracts of nine or more uridines in the human transcriptome, underlining the importance of U-tract length in determining hnRNP C binding to pre-mRNAs (Figure S1B, top).

**Figure 1:**
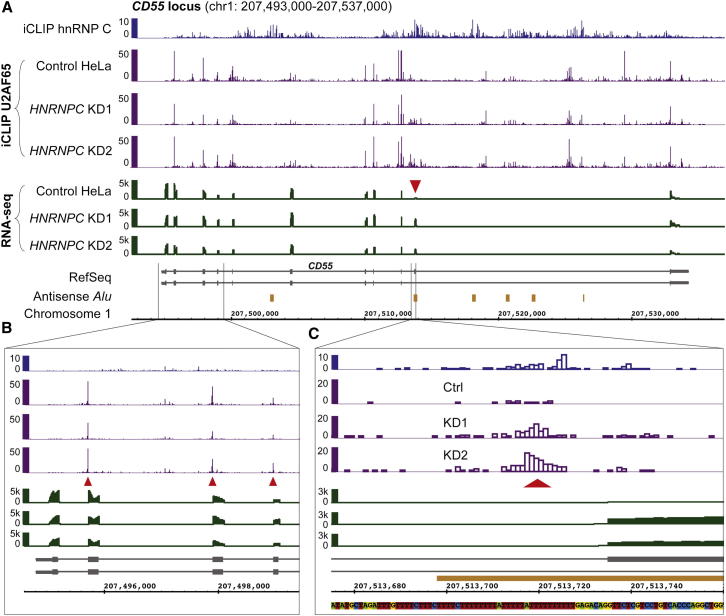
Examples of U2AF65 and hnRNP C Binding at the 3′ Splice Sites of Constitutive or hnRNP-C-Repressed Exons within the *CD55* Gene (A) Genome browser view of the *CD55* gene displaying the iCLIP data (crosslink events per nucleotide) of hnRNP C (blue) and U2AF65 (purple) as well as the RNA-seq data (overlapping reads per nucleotide; green) from control and *HNRNPC* knockdown HeLa cells. The red arrowhead marks the hnRNP-C-repressed alternative *Alu* exon. RefSeq transcript annotations (gray) and *Alu* elements in antisense orientation to the shown strand (orange) are depicted below. (B) Enlargement of the genomic region containing the 5′ UTR and the first four exons. Red arrowheads mark U2AF65 peaks at 3′ splice sites. (C) Enlargement of the region around the 3′ splice site of the hnRNP-C-repressed *Alu* exon (marked in A) including the underlying genomic sequence. The red arrowhead marks the site of increased U2AF65 occupancy in the *HNRNPC* knockdown. See also Figures S1 and S2.

**Figure S2:**
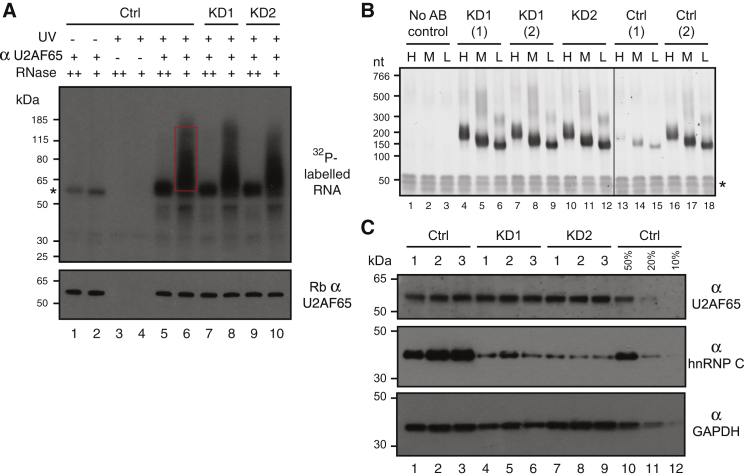
The Protein Abundance and RNA-Binding Ability of U2AF65 Are Not Affected by the *HNRNPC* Knockdown, Related to Figure 1 (A) The total amount of crosslinking of U2AF65 to RNA is not altered in the absence of hnRNP C. Analysis of crosslinked U2AF65-RNA complexes using denaturing gel electrophoresis and membrane transfer. Protein extracts were prepared from UV-crosslinked (UV+) control HeLa cells (Ctrl) and *HNRNPC* knockdown cells (KD1 and KD2), and RNA was partially digested using low (+) or high (++) concentrations of RNase. U2AF65-RNA complexes were immuno-purified with a mouse antibody against U2AF65 (α U2AF65). To allow visualization of the protein-RNA complexes, the 5′ ends of the RNAs were radioactively labeled. The complexes were size-separated using denaturing gel electrophoresis and transferred to a nitrocellulose membrane. The upper panel shows the autoradiograph of this membrane. U2AF65-RNA complexes are shifted upward from the size of the protein (53 kilo Dalton, kDa; lane 6: the red box indicates the region that was extracted for subsequent analyses). This shift is focused when high RNase concentrations are used (lane 5). A similar pattern with comparable intensity is observed for the *HNRNPC* knockdown cells (lanes 7-10), indicating that crosslinking is not generally affected. As a control, no signal is observed in experiments where the antibody was omitted during immunoprecipitation (lanes 3 and 4). Importantly, when omitting UV irradiation, no shifted U2AF65-RNA complexes can be observed. The remaining radioactive signal at the size of U2AF65 (marked by ^∗^) in these samples indicates that part of the protein is labeled under the used conditions. The lower panel shows the Western blot analysis of the same immunoprecipitations with a rabbit antibody against U2AF65 (Rb α U2AF65). (B) Analysis of PCR amplified iCLIP cDNA libraries using gel electrophoresis. RNA recovered by proteinase K digestion from the nitrocellulose membrane as indicated in (A) was reverse transcribed and size-selected using denaturing gel electrophoresis (not shown). Three of the cDNA size fractions (H: 100-170 nt; M: 85-100 nt; L: 75-85 nt) were further processed and PCR amplified (17 cycles of amplification) to obtain the iCLIP libraries. In the gel image, PCR products of different sizes can be observed, according to the size of the input fractions from control HeLa cells (Ctrl, lanes 13-18) and *HNRNPC* knockdown samples (KD1 and KD2, lanes 4-12). When no antibody was used in the immunoprecipitation, no signal is observed (lanes 1-3). Positions of a size standard are given on the left, and the position of the PCR primers is indicated by an asterisk. (C) The protein abundance of U2AF65 is not altered in the *HNRNPC* knockdown. Western blot analyses with antibodies against U2AF65, hnRNP C and GAPDH (indicated on the right) comparing the protein abundances in control HeLa cells (Ctrl, lanes 1-3) and *HNRNPC* knockdown cells (KD1 and KD2, lanes 4-9) in triplicates. The *HNRNPC* knockdown efficiency was estimated to about 20% based on comparison with Ctrl lanes containing 50%, 20% and 10% of input material (lanes 10-12). A protein size marker in kDa is indicated on the left.

**Figure S3:**
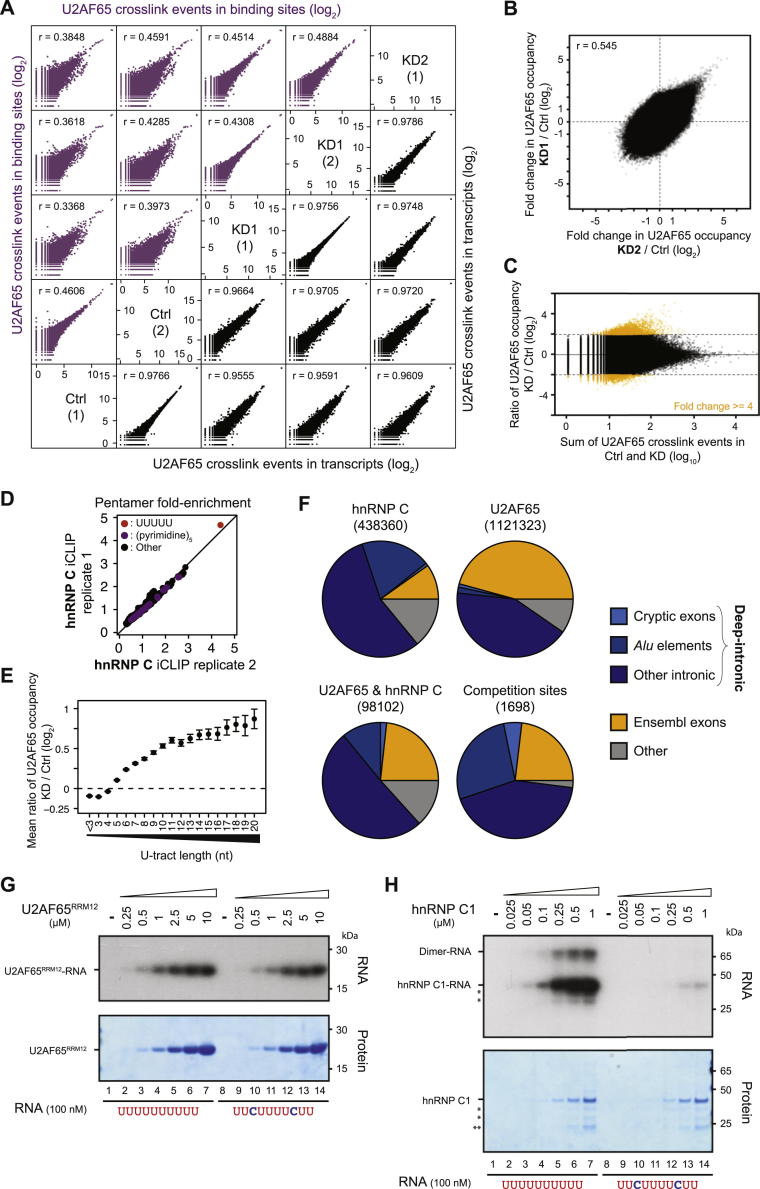
hnRNP C Competes with U2AF65 Binding at Long U-Tracts and Cryptic Exons, Related to Figure 2 (A) Scatter plots comparing the U2AF65 iCLIP crosslink events within binding sites (purple) or Ensembl transcripts (black) for all replicate experiments from control HeLa cells (Ctrl) and *HNRNPC* knockdown cells (KD1 and KD2). Sample types and replicate numbers (in brackets) are given along the diagonal. The Spearman’s rank correlation (r) for each pair is given in the upper left corner of the respective panel. (B) The changes in U2AF65 occupancies on individual binding sites are highly correlated between both knockdowns. Scatter plot comparing the fold changes in U2AF65 occupancy in the two different *HNRNPC* knockdowns (KD1 and KD2). The occupancy was calculated by normalizing the number of crosslink events within each binding site by the total number of crosslink events within the corresponding gene. The Pearson’s product-moment correlation (r) is given in the upper left corner. (C) U2AF65 shows significantly changed occupancy on a large number of binding sites in the *HNRNPC* knockdown. Plot showing the ratio of U2AF65 occupancy from the combined *HNRNPC* knockdowns (KD) over Ctrl against the sum of U2AF65 crosslink events in all samples. The 5,500 binding sites that show an at least 4-fold change in U2AF65 occupancy are shown in yellow. The dashed lines mark a 4-fold change in either direction. (D) hnRNP C preferentially crosslinks to UUUUU pentamers. Scatter plot comparing the pentamer fold-enrichment at crosslink sites from two hnRNP C iCLIP replicate experiments. hnRNP C prefers the pentamer UUUUU (red), but shows no particular enrichment for further pyrimidine combinations (purple) or other pentamers (black). (E) U2AF65-binding sites that overlap with a long U-tract show increased U2AF65 occupancies in the *HNRNPC* knockdown. The average ratio of U2AF65 occupancy from *HNRNPC* knockdown (KD) over control HeLa cells (Ctrl) is shown for U2AF65-binding sites that overlap with U-tracts of varying lengths. Error bars indicated the 95% confidence interval of the mean. (F) Sites of hnRNP C-U2AF65 competition are strongly enriched at deep-intronic locations (p value < 0.001 compared with all other U2AF65-binding sites, hypergeometric test), in particular at cryptic exons and within *Alu* elements. Pie charts showing the fraction of binding sites located at annotated Ensembl exons as well as at deep-intronic positions (subdivided into positions at cryptic exons, within *Alu* elements and other). Graphs are shown from left to right for: all hnRNP C, all U2AF65 binding sites, U2AF65 binding sites that overlap with hnRNP C binding, and U2AF65 binding sites that show an at least 4-fold increase in occupancy in the *HNRNPC* knockdown and overlap with hnRNP C (competition sites). (G) Recombinant U2AF65^RRM12^ shows comparable crosslinking to the wild-type (U_10_) and mutant (U_2_CU_4_CU_2_) RNA oligonucleotides that resemble the upstream U-tract of the *Alu* exon in the *NUP133* minigene (Figure S6). Increasing concentrations of U2AF65^RRM12^ (21 kDa, concentration indicated above in μM) were incubated with radioactively labeled wild-type or mutant RNA oligonucleotide (100 nM), UV crosslinked and analyzed by denaturing gel electrophoresis. The radioactive signal from the RNA crosslinked to the protein can be observed in the autoradiograph (U2AF65^RRM12^-RNA, top panel). Coomassie staining of the same gel serves as loading control (lower panel). (H) Recombinant hnRNP C1 crosslinking is drastically reduced to the mutant RNA oligonucleotide. Experimental setup as in (G) but using increasing concentrations of hnRNP C1 (33 kDa, concentration indicated above). Note that in addition to the signal derived from hnRNP C1 crosslinked to RNA (hnRNP C1-RNA), an additional crosslinking signal is visible at about 65 kDa. This signal is likely derived from two hnRNP C1 proteins crosslinked to one RNA molecule and therefore labeled as dimer-RNA. Impurities are indicated by asterisks on the left (^∗^ C-terminal truncations of hnRNP C1; ^∗∗^ GST).

hnRNP C shows widespread binding across introns (Figures 1 and S1A). In addition to this broad pattern, the protein shows specific binding to the polypyrimidine tracts of alternative exons that it represses (Figure S1C; repressed exons determined from RNA-sequencing [RNA-seq] data, see below). To explore the effects of this binding, we performed iCLIP experiments with the splicing factor U2AF65 that associates with polypyrimidine tracts to enable exon inclusion (Figures S2A and S2B). This yielded a total of 12 million crosslink events, corresponding to 518,794 binding sites (Table S1). The replicate data sets revealed very consistent binding locations and normalized occupancies within each site, indicating the high reproducibility of the quantitative information contained within the iCLIP data (Figure S3A).

In contrast to the extended binding pattern of hnRNP C, U2AF65 displays more restricted binding, with a strong preference for the regions directly upstream of 3′ splice sites (Figures 1, S1A, and S1D). We detect U2AF65 binding at 58% of all actively used 3′ splice sites in HeLa cells, underlining its crucial role in splice-site recognition. Intriguingly, U2AF65 binding at the 3′ splice sites of all exons (Figure S1D) coincides with the peak of hnRNP C binding at repressed exons (Figure S1C), suggesting that the two proteins might compete for pre-mRNA binding.

### hnRNP C Competes with U2AF65 Binding

To assess competition between hnRNP C and U2AF65, we performed U2AF65 iCLIP experiments in *HNRNPC* knockdown HeLa cells (Figures S2A and S2B). Independent *HNRNPC* knockdowns with two different siRNAs affected neither U2AF65 protein levels nor the protein’s general ability to bind RNA (Figures S2A and S2C). The experiments in the knockdown cells produced 15 million highly reproducible crosslink events; combined with the data from the control samples, this yielded a total of 1.1 million U2AF65-binding sites (Table S1). To compare differences in U2AF65 binding between conditions, we corrected for changes in transcript levels by normalizing the numbers of crosslink events. The changes in U2AF65 binding in the two independent *HNRNPC* knockdowns were highly correlated, allowing us to combine both data sets for the remaining analyses (referred to as KD; r = 0.545, Pearson’s product-moment correlation; Figure S3B).

**Figure 2:**
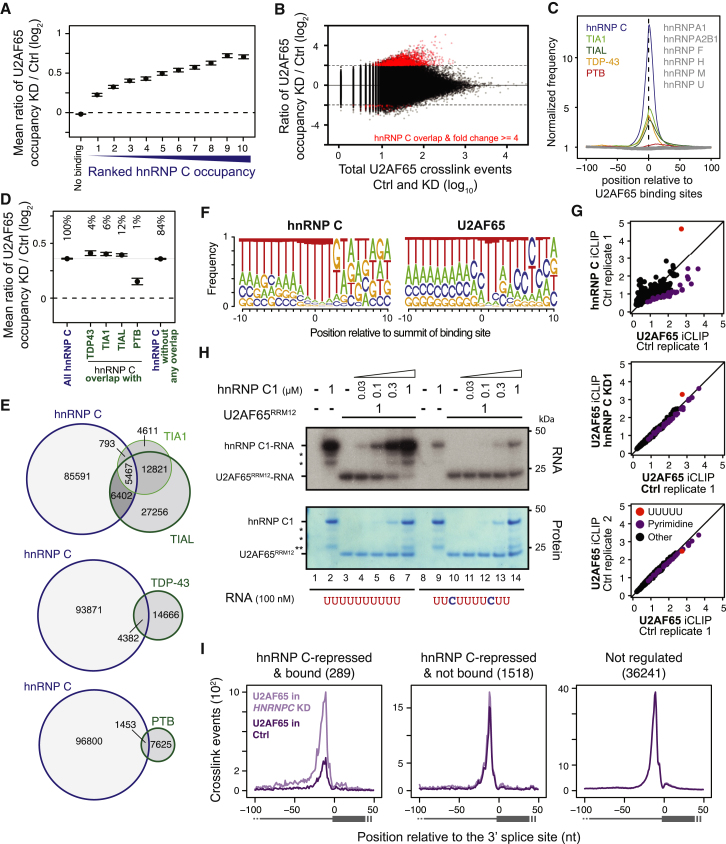
hnRNP C and U2AF65 Compete for Binding on U-Tracts and at Regulated Exons (A) The average ratio of U2AF65 occupancies from knockdown (KD) over control is shown for U2AF65-binding sites that do not overlap with hnRNP C (no binding) or that overlap with hnRNP-C-binding sites within different ranks of hnRNP C occupancy (i.e., rank 10 contains the 10% strongest hnRNP-C-binding sites). Error bars indicate the 95% confidence interval of the mean. (B) Plot showing the ratio of U2AF65 occupancies against the total number of U2AF65 crosslink events for individual binding sites. Binding sites that show an at least 4-fold change in occupancy and overlap with hnRNP C binding are depicted in red. (C) Plots depicting the frequency of overlapping binding sites of ten other RNA-binding proteins around the summit of U2AF65-binding sites (position 0) that overlap with hnRNP C. Proteins that show increased crosslinking are colored. (D) Plot as in (A) showing U2AF65-binding sites that overlap with hnRNP C compared to sites that overlap with both hnRNP C and TIA1, TIAL, TDP-43, or PTB (indicated below) and sites that overlap with only hnRNP C and none of the other proteins. The percentage of shared hnRNP-C-U2AF65-binding sites within the different categories is indicated above. (E) Weighted Venn diagrams depicting the overlap of U2AF65-binding sites that are bound by hnRNP C (blue) and/or either TIA1/TIAL, TDP-43, or PTB (green). Absolute numbers are given within each segment. (F) Weblogos showing the relative nucleotide frequency around the summits (position 0) of hnRNP-C- and U2AF65-binding sites. (G) Plots comparing the pentamer fold-enrichment around crosslink sites from replicate experiments with hnRNP C and U2AF65 from control and *HNRNPC* knockdown HeLa cells. The three panels compare iCLIP data from (i) experiments with both proteins from untreated HeLa cells (Ctrl; left), (ii) replicate experiments with U2AF65 from Ctrl cells (middle; see also Figure S3D), and (iii) experiments with U2AF65 from *HNRNPC* knockdown (KD1) and Ctrl cells (right). (H) Autoradiograph from an in vitro UV crosslinking assay using recombinant hnRNP C1 (33 kDa) and U2AF65^RRM12^ proteins (21 kDa). A stable amount of U2AF65^RRM12^ plus increasing concentrations of hnRNP C1 (indicated above in μM) were UV crosslinked to radioactively labeled wild-type (U_10_, lanes 1–7) and mutant (U_2_CU_4_CU_2_, lanes 8–14) RNA oligonucleotides (100 nM) and analyzed by denaturing gel electrophoresis. Radioactive signals of RNA crosslinked to hnRNP C1 or U2AF65^RRM12^ are marked on the left. Asterisks indicate C-terminal hnRNP C1 truncations (^∗^) and GST (^∗∗^). Coomassie staining of the same gel (bottom) serves as loading control. Note that there is an additional hnRNP C1 signal likely representing an hnRNP C1 dimer, which is only shown in Figure S3H. (I) RNA maps showing the total number of crosslink events of U2AF65 in control HeLa (light purple) and *HNRNPC* knockdown cells (dark purple) relative to the 3′ splice sites of all exons that (i) are repressed and bound by hnRNP C (left), (ii) are repressed but not bound by hnRNP C (middle), and (iii) are not subject to any regulation in the *HNRNPC* knockdown (fold change < 1.1; right). The number of exons in each category is indicated above. See also Figure S3.

Loss of hnRNP C has a dramatic impact on U2AF65 binding: thousands of sites display increased U2AF65 occupancy, with over 3,000 sites showing at least 4-fold increases (Figure S3C). Importantly, these changes are largest at sites that directly overlap with hnRNP C binding (p value < 10^−16^, Student’s t test; Figure 2A), suggesting that U2AF65 gains access to sites that are normally occupied by hnRNP C. In particular, 1,698 (51%) of the ∼3,000 most upregulated sites coincide with an hnRNP-C-binding site (Figure 2B; p value < 10^−15^ compared with unchanged sites, Fisher’s exact test). Significantly, the changes in U2AF65 occupancies increase with the strength of hnRNP C binding (Figure 2A), in line with the characteristics of competitive binding. Moreover, only 4% or 7% of binding sites with decreased or unchanged U2AF65 occupancy coincide with hnRNP C binding, respectively. These results indicate that hnRNP C blocks U2AF65 from a large number of binding sites in the transcriptome.

A number of hnRNP proteins, including hnRNP H (Heiner et al., 2010), PTB (hnRNP I; Saulière et al., 2006), and hnRNP A1 (Tavanez et al., 2012), have previously been described to compete with, or proofread, U2AF65 binding. To test whether these and other RNA-binding proteins function together with hnRNP C, we examined published binding data for seven hnRNP proteins (hnRNP A1, A2B1, F, H, M, U, and PTB; Huelga et al., 2012; Xue et al., 2009). We also included TIA1, TIAL, and TDP-43, which recognize U-rich motifs (Tollervey et al., 2011; Wang et al., 2010). TIA1, TIAL, TDP-43, and PTB, but none of the other proteins, display noticeable crosslinking at loci containing both U2AF65 and hnRNP C binding (Figure 2C). We tested whether their presence affects competitive binding by assessing the changes in U2AF65 occupancies in the *HNRNPC* knockdown. As expected, most of the increase in U2AF65 binding (at sites overlapping with hnRNP C) can be explained by the loss of hnRNP C alone (84%; Figure 2D). Sites additionally containing TIA1, TIAL, or TDP-43 binding show a slightly bigger increase. In contrast, sites overlapping with PTB show a reduced shift (Figure 2D), suggesting that PTB and hnRNP C might act redundantly. However, because we observe these combinatorial effects only at a minor fraction of sites (16% of all shared U2AF65-hnRNP-C-binding sites; Figure 2E), we conclude that hnRNP C alone is sufficient to compete with U2AF65 in the majority of cases.

A key feature of the competition between hnRNP C and U2AF65 is their overlapping, but differing, sequence specificity: both proteins bind uridines, but U2AF65 can also recognize cytidines (Figure 2F; Görlach et al., 1994; König et al., 2010; Norton, 1994; Singh et al., 2000; Swanson and Dreyfuss, 1988). A comparison of pentameric sequences within binding sites shows that U2AF65 associates with diverse uridine- and cytidine-containing pentamers, contrasting hnRNP C’s selective preference for continuous uridines (Figures 2G and S3D). In the *HNRNPC* knockdown, we observe a specific increase in U2AF65 binding only to the uridine pentamer (Figure 2G), indicating that U-tracts that are otherwise protected by hnRNP C become accessible. Consistently, the biggest changes in U2AF65 binding occur at long U-tracts (Figure S3E). These observations suggest that the competition between the two proteins occurs at a specific subset of U2AF65-binding sites, namely at long U-tracts, which constitute prime hnRNP-C-binding sites.

### hnRNP C Blocks U2AF65 from Continuous U-Tracts In Vitro

To test whether the competition is direct, we performed in vitro UV-crosslinking assays (Warf et al., 2009). For this, we used a recombinant full-length hnRNP C1 protein and a fragment of U2AF65 containing the first two RRM domains (U2AF65^RRM12^), which was previously shown to retain the binding characteristics of the full-length protein (Mackereth et al., 2011). We first tested the binding of both proteins individually to two RNA oligonucleotides, resembling a high-affinity hnRNP-C-binding site (U_10_), as well as a modified version carrying two cytidines at positions 3 and 8. Whereas U2AF65^RRM12^ shows comparable binding to both RNAs, hnRNP C1 binding is drastically impaired by the two interspersed cytidines (Figures S3G and S3H), in line with the divergent sequence preferences of the two proteins.

We next assessed the direct competition between both proteins by adding increasing concentrations of hnRNP C1 to the U2AF65^RRM12^-binding reaction. U2AF65^RRM12^ binding to the U_10_ RNA decreases with increasing amounts of hnRNP C1 and is almost completely abolished at equimolar concentrations (Figure 2H). Conversely, U2AF65^RRM12^ binding to the cytidine-containing RNA remains largely unaffected in the presence of hnRNP C1. These results demonstrate that hnRNP C alone is sufficient to displace U2AF65 from continuous U-tracts. Notably, this competition is alleviated by interspersed cytidines, allowing strong U2AF65 binding in the presence of hnRNP C.

### The hnRNP C-U2AF65 Competition Leads to Exon Repression

**Figure S4:**
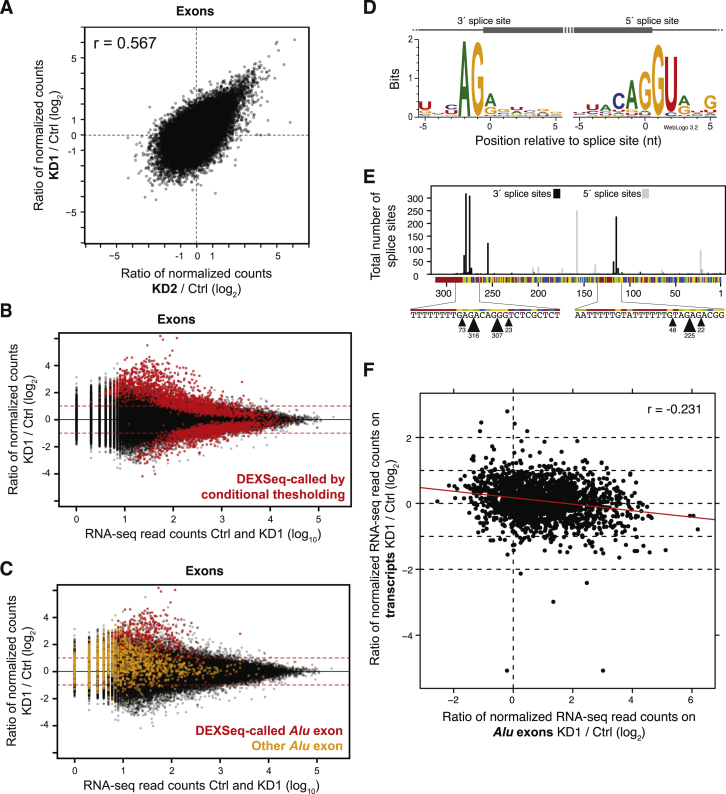
RNA-Seq Identifies a Large Number of hnRNP-C-Repressed *Alu* Exons, Related to Figure 3 (A) The detected splicing changes show a strong correlation between both *HNRNPC* knockdowns. Scatter plot comparing the fold changes (log_2_) in normalized exon expression (*HNRNPC* knockdown over control HeLa cells) from both *HNRNPC* knockdowns (KD1 and KD2). The Pearson’s product-moment correlation (r) is given in the upper left corner. (B) The majority of changed exons show higher inclusion in the *HNRNPC* knockdown. Plot indicating the fold change (log_2_) in normalized exon expression in KD1 against the sum of RNA-seq reads detecting the exon in KD1 and control samples. Exons that are called by DEXSeq are indicated in red (p values used for conditional thresholding p_a_ = 0.01 and p_b_ = 0.05). (C) The *Alu* exons show widespread upregulation in the *HNRNPC* knockdown. Plot as in (B). DEXSeq-called *Alu* exons are highlighted in red, and all other *Alu* exons are depicted in yellow. (D) The *Alu* exons arise from canonical splice sites. Weblogos indicating the consensus sequence at the 3′ and 5′ splice sites of the *Alu* exons. (E) Bar diagram summarizing the usage of different 3′ (black) and 5′ (gray) splice sites in the *Alu* consensus sequence (color coding as for enlarged sequence below). The region around the most commonly used 3′ splice sites is enlarged below (black arrowheads; the number of exons using each splice site is given below the arrowhead). (F) Scatter plot comparing changes in *Alu* exon inclusion (*x* axis) with changes in the corresponding transcript levels. The linear regression line (red) indicates the negative correlation between both data sets (Pearson’s product-moment correlation r = −0.231).

Our iCLIP and in vitro binding data demonstrate that hnRNP C blocks U2AF65 from a large number of binding sites. To investigate how this competition influences splicing, we performed RNA-seq experiments using the same two *HNRNPC* knockdowns as well as control HeLa cells (Table S1). We first monitored changes in gene expression using the DESeq software, identifying 4,880 and 4,875 genes that showed significant differential expression in KD1 and KD2, respectively (adjusted p value < 0.01). Using Cufflinks, we then determined transcript structures de novo to detect all expressed exons in the knockdown and control samples (Trapnell et al., 2010). We identified changes in splicing patterns using the DEXSeq software (Anders et al., 2012) and observed a good correlation in splicing changes between both knockdowns; this indicates that the changes arise as a consequence of hnRNP C depletion rather than off-target effects of the siRNAs used (r = 0.567, Pearson’s product-moment correlation; Figure S4A). By combining the knockdown data sets, we obtained a high-confidence set of 3,052 differentially expressed exons, including 1,807 and 1,245 that are repressed and enhanced by hnRNP C, respectively (Table S2; Figure S4B).

A total of 289 (16%) repressed exons harbor an hnRNP-C-binding site less than 30 nucleotides upstream of their 3′ splice site (14-fold enrichment compared with all other exons; p value < 10^−15^, Fisher’s exact test), compared with only 1%–2% of enhanced or unchanged exons. Using an RNA map depicting changes in U2AF65 binding, we find a clear 3-fold increase in U2AF65 occupancy upon *HNRNPC* knockdown at exons that are bound and repressed by hnRNP C (Figure 2I). In dramatic contrast, exons that are either not regulated or not bound by hnRNP C display no change in U2AF65 binding (Figure 2I). We conclude that competition with U2AF65 constitutes the mechanism of hnRNP-C-mediated repression of exons with proximal hnRNP C binding, whereas the remaining exons might be regulated via distal hnRNP-C-binding sites or other effects that do not alter U2AF65 binding. An example of a competitive event at the 3′ splice site can be seen at the alternative exon of the *CD55* gene: *HNRNPC* knockdown leads to a strong increase in U2AF65 binding, accompanied by significantly elevated exon inclusion (Figure 1C). In summary, these observations indicate that hnRNP C represses alternative exons by directly interfering with U2AF65 recognition.

### hnRNP C Prevents the Aberrant Exonization of *Alu* Elements

The observations so far have explained the function of hnRNP C at known alternative exons; however, it is clear that the vast majority of hnRNP C binding occurs at “deep” intronic regions without exon annotations (Figure S1A). Strikingly, we find that hnRNP C binding in these regions also blocks U2AF65 activity: in fact, 75% of the U2AF65-binding sites that display strongest competition with hnRNP C are located more than 200 nucleotides away from any Ensembl-annotated exon (Figure S3F), suggesting that hnRNP C prevents recognition of cryptic splicing signals. This is confirmed in the RNA-seq data, which show that 41% of hnRNP-C-repressed exons do not overlap with existing annotations in the Ensembl database (Table S2). This indicates that hnRNP C prevents the aberrant inclusion of cryptic exons that are normally excluded from transcripts.

A major source of cryptic exons are *Alu* elements. They are the most abundant transposable elements in the human genome, present in over 50% of all introns (Deininger, 2011). They contain two arms separated by a poly(A) linker sequence and followed by a poly(A) tail. When integrated into genes in the antisense orientation, the poly(A) sequences are transcribed as U-tracts (referred to as the upstream and the linker U-tract) that can serve as polypyrimidine tracts to promote recognition of the cryptic splice sites that exist within the *Alu* elements (Deininger, 2011). During primate evolution, many *Alu* elements evolved into genuine exons in a process called “exonization.” Among them is the Ensembl-annotated *Alu* exon in the *CD55* gene, whose inclusion is clearly regulated via competition between hnRNP C and U2AF65 (Figure 1C).

**Figure 3:**
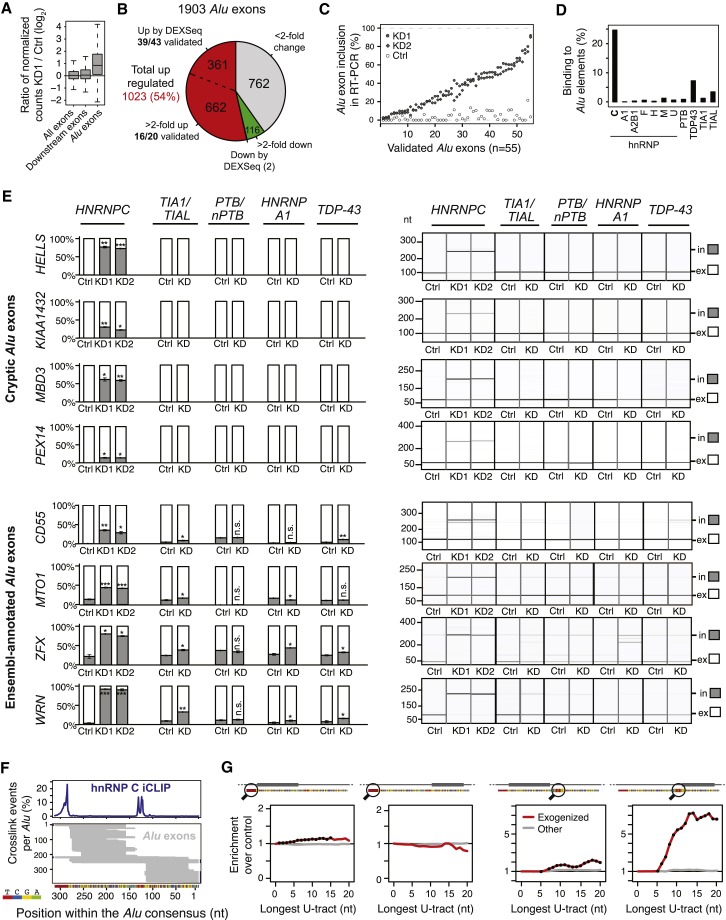
The *HNRNPC* Knockdown Leads to Widespread Exonization of Antisense *Alu* Elements (A) Box plots summarizing the change in normalized expression of *Alu* exons compared to downstream non-*Alu* exons as well as all exons. (B) Pie chart summarizing the regulation of all 1,903 *Alu* exons detect from our RNA-seq data. Upregulated and downregulated exons are further subdivided into those called by DEXSeq or displaying a more than 2-fold change in the *HNRNPC* knockdown. (C) Plot depicting the mean inclusion levels in control HeLa cells (open diamonds) and both *HNRNPC* knockdowns (KD1, filled circles; KD2, filled diamonds) of 55 *Alu* exons that were measured by RT-PCR (Data S1A and S1B). (D) Bar chart showing the percentage of binding sites of hnRNP C and ten other RNA-binding proteins (indicated below) that overlap with antisense *Alu* elements. (E) Semiquantitative RT-PCR analyses of four cryptic and four Ensembl-annotated *Alu* exons (indicated on the left) upon knockdown of *HNRNPC* (two independent knockdowns, KD1 and KD2), *TIA1/TIAL*, *PTB/nPTB*, *HNRNPA1,* and *TDP-43* (labeled as KD with the respective gene[s] indicated above) as well as in control HeLa cells (Ctrl). Known target exons of these proteins can be found in Data S1C. (Right) Gel views of capillary electrophoresis of the PCR products with the fragments including (in) or excluding (ex) the *Alu* exon marked on the right. (Left) Bar diagrams depicting the mean inclusion (gray) and exclusion (white) level in each sample. Asterisks indicate the significance level (Student’s t test) relative to control: n.s., nonsignificant; ^∗^p value < 0.05; ^∗∗^p value < 0.001; ^∗∗∗^p value < 0.0001. Error bar represents SDM; n = 3. (F) Schematic representations of hnRNP C crosslink events per nucleotide (top) and of *Alu* exon locations (bottom) along the *Alu* consensus sequence. Exons that extend beyond the *Alu* element end with a blue dash. (G) Plots depicting the ratio of the cumulative frequencies of U-tracts of a given length (e.g., at least five uridines) in exonized *Alu* elements (red line) compared to nonexonized *Alu* elements within the same genes. Analyses are separately shown for *Alu* exons from the first or second arm of the *Alu* element (gray rectangle above) as well as for the upstream and linker U-tracts (magnifier icon). Nonexonized *Alu* elements of all other genes (gray line) serve as control. Black dots, p value < 0.05 (Pearson’s chi-square test). See also Figures S4 and S5, Data S1, and Table S3.

Our RNA-seq data document the presence of a large number of *Alu*-derived exons. In addition to 585 Ensembl-annotated *Alu* exons, we find 1,318 cryptic exons that originated from *Alu* elements, yielding a total of 1,903 *Alu* exons for further analysis (Table S2). hnRNP C depletion leads to a dramatic global increase in *Alu* exon inclusion (Figures 3A and S4C): we detect a total of 1,023 upregulated *Alu* exons that are either identified by the DEXSeq software (361 exons) or display a more than 2-fold change in at least one knockdown (662 exons; Figures 3B and S4C; Table S2). We used these two thresholds because many *Alu* exons are expressed at low levels, leading to lower statistical power for the DEXSeq analysis.

We confirmed the splicing changes by semiquantitative RT-PCR, validating 39 out of 43 (91%) DEXSeq-called *Alu* exons and 16 out of 20 (80%) *Alu* exons with more than 2-fold change (Figure 3C; Data S1; Table S3). We conclude that more than 1,000 *Alu* exons show a considerable increase in inclusion upon hnRNP C depletion (Figure 3B). Most *Alu* exons are barely detectable in the control samples (Figure 3C), emphasizing how efficiently they are suppressed under normal conditions. In contrast, these same exons display up to 90% inclusion in the knockdown. Together, these results demonstrate that hnRNP C safeguards the transcriptome from aberrant and potentially detrimental expression of cryptic exons, most particularly those originating from *Alu* elements.

Finally, we tested whether the repression of *Alu* exons is specific to hnRNP C. Among ten other RNA-binding proteins tested, TDP-43 and TIAL show most binding to *Alu* elements (7.2% and 3.4% of binding sites, respectively; Figure 3D), but far less than hnRNP C (25%; see below). We also tested the inclusion of four cryptic and four Ensembl-annotated *Alu* exons upon depletion of TDP-43, TIA1/TIAL, as well as hnRNP A1 and PTB/nPTB. None of these knockdowns triggers exonization of the cryptic *Alu* exons (Figure 3E; Data S1C). For the Ensembl-annotated *Alu* exons, we find some regulation by all proteins. However, all of these changes are small compared to the impact of hnRNP C depletion, and we cannot exclude indirect effects; for instance, *TIA1/TIAL* knockdown was previously described to alter the splicing pattern of *HNRNPC* (Wang et al., 2010). In summary, we conclude that the suppression of *Alu* exons is specifically and primarily achieved by hnRNP C.

### The U-Tracts Facilitate Strong hnRNP C Binding to *Alu* Elements

The impact of hnRNP C function extends far beyond the 1,000 *Alu* exons that we detect as repressed by hnRNP C. hnRNP C binds extensively to antisense *Alu* elements in the transcriptome: we detect binding to 72,625 intronic antisense *Alu* elements, comprising 21% of all antisense *Alu* elements in the transcriptome (compared with only 0.03% of sense *Alu* elements). In fact, 25% of all hnRNP-C-binding sites occur within intronic *Alu* elements, underlining that repression of *Alu* exonization is a major role of hnRNP C. Within the *Alu* elements, hnRNP C recognizes the upstream and linker U-tracts, where its binding coincides with cryptic *Alu* exons originating from both arms (Figures 3F, S4D, and S4E). Together, these observations suggest that the long continuous U-tracts of the *Alu* elements attract strong hnRNP C binding and serve as a critical interface to control *Alu* exonization.

**Figure S5:**
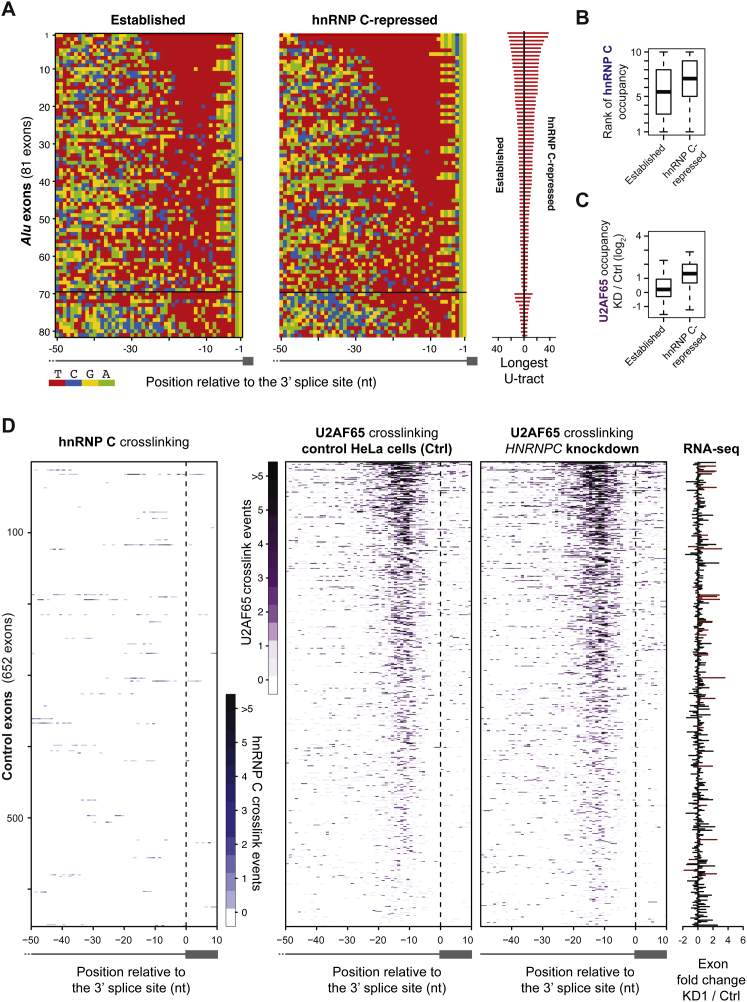
Polypyrimidine Tracts of Established Bona Fide *Alu* Exons Exhibit Shorter Contiguous U-Tracts, Related to Figures 3 and 4 (A) Established *Alu* exons accumulate point mutations that disrupt the long U-tracts. Heatmaps visualizing the polypyrimidine tract of *Alu* exons originating from the first (top) and second (bottom) arm of *Alu* elements (separated by a horizontal black line). Each line represents the polypyrimidine tract of an individual *Alu* exon, with the different colors indicating the four bases (T: red; C: blue; G: yellow; A: green). In the left panel, the 81 pyrimidine tracts of established bona fide *Alu* exons are shown that are highly included and show a less than 1.5-fold change in either direction, while the right panel shows the same regions of a randomly selected subset of hnRNP-C-repressed *Alu* exons. The exons were sorted according to the longest contiguous U-tract, as indicated in the bar chart on the right. (B) Established *Alu* exons harbor weaker hnRNP-C-binding sites. Box plot comparing the distribution of ranks of hnRNP-C-binding sites in front of 81 established or 81 randomly selected hnRNP-C-repressed *Alu* exons (p value < 0.1, Student’s t test). (C) U2AF65-binding sites at established *Alu* exons show hardly any change in occupancy upon knockdown of *HNRNPC*. Box plot depicting the ratio of U2AF65 occupancies from *HNRNPC* knockdown (KD) and control HeLa cells (Ctrl) of U2AF65-binding sites in front of 81 established or 81 randomly selected *Alu* exons (p value < 10^−9^, Student’s t test). (D) Control exons show little hnRNP C crosslinking and no increase in U2AF65 crosslinking in the *HNRNPC* knockdown. Heatmaps comparing the crosslinking of hnRNP C and U2AF65 relative to the 3′ splice site of the non-*Alu* exons downstream of the *Alu* exons shown in Figure 4. Each row corresponds to one of 652 exons. The left panel shows the crosslink events of hnRNP C at each nucleotide (nt) depicted in different shades of blue as indicated aside. The middle and right panels visualize U2AF65 crosslinking (different shades of purple) in control HeLa (Ctrl) and *HNRNPC* knockdown cells (KD). The bar diagram on the right (RNA-seq) shows the corresponding fold change in exon inclusion (KD1 / WT). Fold change values found to be significant according to the conditional thresholding approach are shown in red.

We further assessed the importance of the U-tracts in *Alu* elements by performing an evolutionary analysis measuring the strength of selection. Compared with unexonized *Alu* elements, *Alu* elements that give rise to hnRNP-C-repressed exons show a remarkable tendency to preserve and even lengthen U-tracts (Figure 3G). This indicates that stronger hnRNP C binding and hence stronger repression of cryptic *Alu* exons provided a substantial fitness benefit during primate evolution, and that accidental *Alu* exonization imposes a significant cost to survival. In contrast, a separate comparison with 81 established *Alu* exons, which are included in control cells and do not change in the *HNRNPC* knockdown, reveals a trend toward shorter U-tracts and mutations that weaken hnRNP-C-binding (Figures S5A–S5C). These observations indicate that there is overwhelming selection pressure to repress aberrant *Alu* exonization through hnRNP C binding, and that this is relieved in only a very small subset of *Alu* elements that become genuine exons.

### The Competition between hnRNP C and U2AF65 Controls *Alu* Exonization

**Figure 4:**
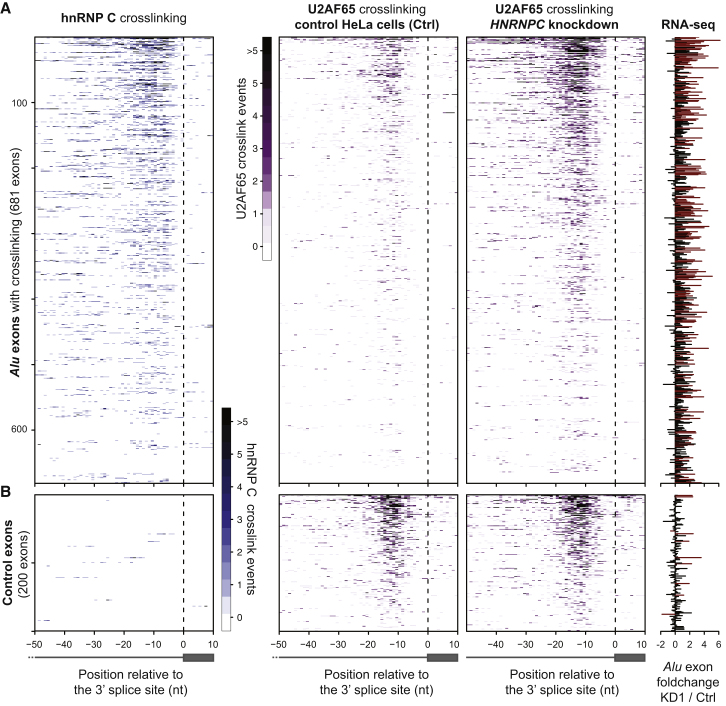
The Competition of hnRNP C with U2AF65 at 3′ Splice Sites Represses *Alu* Exon Inclusion (A) Heatmaps comparing the amount of crosslinking of hnRNP C (left; different shades of blue) and U2AF65 (different shades of purple) in control (middle) and *HNRNPC* knockdown cells (right) relative to the 3′ splice sites of *Alu* exons (indicated by a dashed line). U2AF65 iCLIP data were corrected for differences in library sizes. Each row corresponds to one of 681 *Alu* exons that contain at least five crosslink events within the analyzed region (from −50 nt to +10 nt relative to the first nucleotide of the exon). The bar diagram on the right shows the fold change in *Alu* exon inclusion (KD1 over wild-type; differentially regulated exons according to conditional thresholding are shown in red). (B) Heatmaps as in (A) for a subset of 200 control non-*Alu* exons that lie downstream within the same genes (full set in Figure S5D). See also Figure S5.

To investigate whether hnRNP C interferes with U2AF65 binding to cryptic exons, we compared binding patterns in the knockdown and control samples. We find a 3.3-fold increase in U2AF65 binding at the 3′ splice sites of *Alu* exons in the *HNRNPC* knockdown (Figure 4A). This effect is specific for the *Alu* exons, because downstream control exons remain unaffected (Figures 4B and S5D). This indicates that hnRNP C efficiently blocks U2AF65 from *Alu* elements.

**Figure 5:**
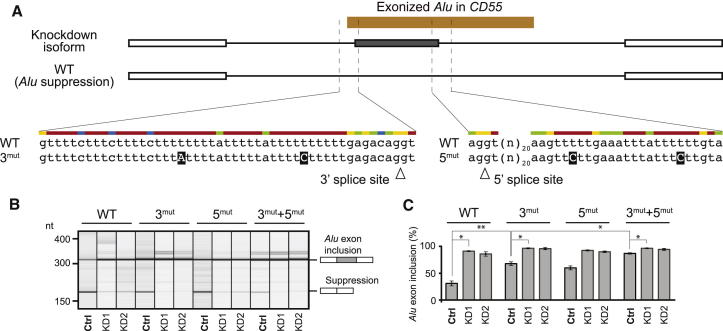
Point Mutations that Impair hnRNP C Binding Promote Inclusion of the *Alu* Exon in the *CD55* Minigene (A) Schematic overview of the minigene including the *Alu* exon (gray square), intronic regions (black lines), and two flanking exons (white squares) from the *CD55* gene. The original sequence (WT) as well as the mutated sequence surrounding the 3′ and 5′ splice sites (3^mut^ and 5^mut^, respectively; splice sites marked by arrowheads) are depicted below. Introduced point mutations are highlighted in black. (B) RT-PCR monitoring inclusion or suppression of the *Alu* exon in the minigenes with wild-type (WT) or mutated sequences (3^mut^, 5^mut^) in *HNRNPC* knockdown (KD1 and KD2) and control HeLa cells (Ctrl). The corresponding capillary electrophoresis data is given in a gel-like representation with *Alu* exon inclusion and suppression indicated schematically on the right. (C) Average *Alu* exon inclusion in percent from three replicate RT-PCR experiments. Lines indicate relevant comparisons with asterisks representing different levels of significance (^∗^p value < 0.05; ^∗∗^p < 10^−3^; Student’s t test). Error bars represent SDM. See also Figure S6 and Table S5.

To confirm that the integrity of the U-tract is important for hnRNP-C-based repression of *Alu* exons, we generated a minigene containing the Ensembl-annotated *Alu* exon within the *CD55* gene (Figures 1 and 5A). Based on our in vitro UV-crosslinking assays, we hypothesized that mutations disrupting the U-tract but preserving the polypyrimidine tract would weaken hnRNP C binding and hence increase exon inclusion. We find that two point mutations in the upstream U-tract are sufficient to elevate inclusion levels in the presence of hnRNP C (Figure 5), confirming that high-affinity hnRNP C binding is critical for efficient competition with U2AF65. Consistent with our previous observation that hnRNP C binding occurs on both sides of regulated exons (König et al., 2010), introduction of additional mutations in the downstream linker U-tract further increases exon inclusion and almost completely abolishes hnRNP C-dependent regulation.

**Figure S6:**
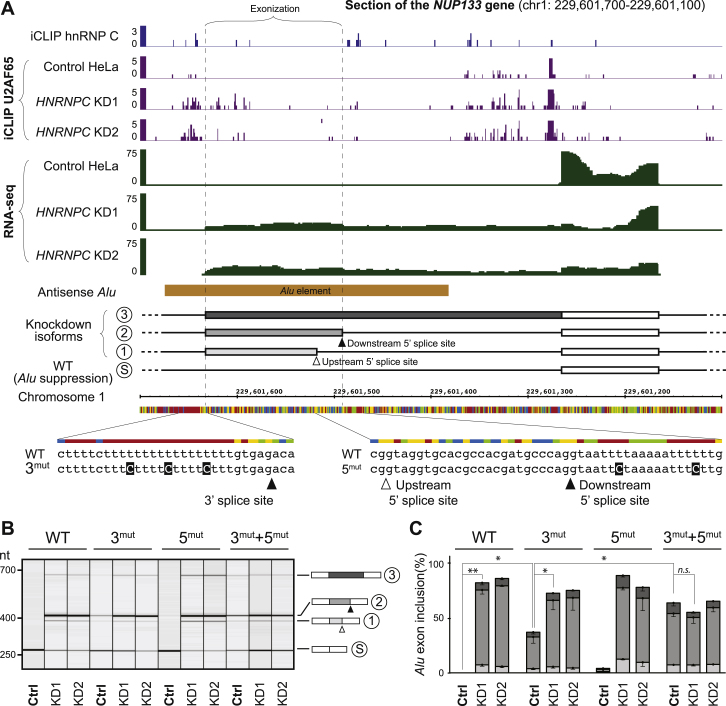
hnRNP C Suppresses the Exonization of an *Alu* Element in the *NUP133* Gene through Competition with U2AF65, Related to Figure 5 (A) Genome browser view including an *Alu* element (orange) plus the downstream exon within the *NUP133* gene on chromosome 1 (229,601,700-229,601,100). hnRNP C iCLIP data (blue) show crosslinking at the upstream and the linker U-tract of the *Alu* element (corresponding thymidines are highlighted in red at the bottom). Little U2AF65 crosslinking (purple) is detected at the U-tracts in control HeLa cells, while strong crosslinking is observed in the *HNRNPC* knockdowns (KD1 and KD2). RNA-seq data (green) show exonization of the *Alu* element in both knockdowns, giving rise to three different *Alu* exon variants. The corresponding isoforms are schematically indicated: *Alu* suppression in isoform S, *Alu* inclusion as a cassette exon in isoform 1 and 2 (differing in the usage of an upstream or downstream 5′ splice site, respectively) and *Alu* inclusion using an alternative 3‘ splice site for the downstream exon (isoform 3). The wild-type (WT) sequence surrounding the 3′ and 5′ splice sites (arrowheads) including the introduced point mutations (3^mut^ and 5^mut^) are shown below. The positions of the point mutations are highlighted in the sequences by black squares. (B) U-to-C transitions promote *Alu* exonization in the presence of hnRNP C. Shown is the gel-like view of capillary electrophoresis of RT-PCR analysis of minigenes containing the *Alu* element in the *NUP133* gene described in (A) with two flanking exons. The wild-type (WT) minigene shows no *Alu* exonization in control HeLa cells (Ctrl) and a significant increase in isoforms 1-3 in the *HNRNPC* knockdown (KD1 and KD2). A mutant minigene containing three T-to-C transitions in the upstream U-tract (3^mut^) shows significant inclusion of isoforms 1-3 in control HeLa cells. Additional T-to-C transitions in the linker U-tract (5^mut^) further elevate the inclusion of isoforms 1-3 in control HeLa cells and prevent any further regulation by hnRNP C (KD1 and KD2). The sizes corresponding to the different analyzed isoforms are schematically indicated on the right. (C) Average *Alu* exon inclusion in percent for three replicate RT-PCR experiments as described in (B). The different isoforms are indicated by shades of gray (light gray: isoform 1; medium gray: isoform 2; dark gray: isoform 3). Lines indicate relevant comparisons with asterisks indicating different levels of significance for changes in the summed inclusion isoforms (^∗^: p value < 0.05; ^∗∗^: < 10^−3^; n.s.: not significant; Student’s t test). Error bars represent SD of the mean; n=3.

To test the effects of similar mutations in cryptic *Alu* exons, we generated another minigene containing the intronic *Alu* element in the *NUP133* gene that exonizes upon *HNRNPC* knockdown (Figure S6A). As observed for *CD55*, introduction of three point mutations in the upstream U-tract is sufficient to promote *Alu* exonization, and two additional mutations in the linker U-tract completely abolish hnRNP C repression (Figure S6). In summary, these experiments demonstrate that the competition between hnRNP C and U2AF65 controls *Alu* exonization, and that weakening hnRNP C binding is sufficient to promote exon inclusion.

### Mutations Disrupting hnRNP-C-Dependent Repression of *Alu* Exons Can Cause Disease

**Figure 6:**
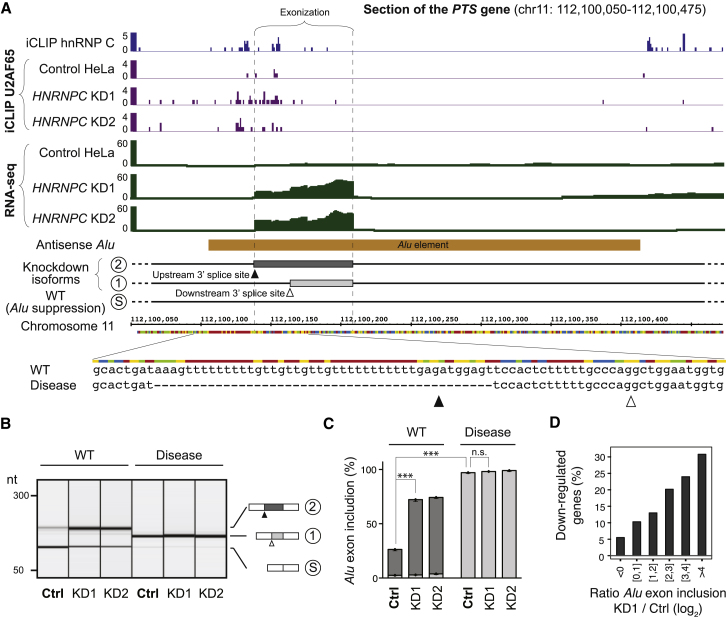
hnRNP C Repression of *Alu* Exonization in the *PTS* Gene Is Relevant for Disease (A) Genome browser view including the disease-relevant *Alu* element (orange) within the *PTS* gene. iCLIP data for hnRNP C (blue) and U2AF65 (purple) from *HNRNPC* knockdown (KD1 and KD2) and control HeLa cells as well as RNA-seq data (green) are shown above. The corresponding isoforms are schematically indicated: *Alu* suppression in isoform S, usage of the downstream 3′ splice site (open arrowhead) in isoform 1 (light gray; this isoform is produced as a result of the disease-associated deletion which removes the upstream 3′ splice site together with the U-tract) and usage of the upstream 3′ splice site (filled arrowhead) in isoform 2. Wild-type sequence (WT) and disease-associated deletion are shown below. (B) Gel-like view of capillary electrophoresis of RT-PCR analyses of minigenes containing the *Alu* element described in (A) with the two flanking exons. The different isoforms are schematically indicated on the right. (C) Average *Alu* exon inclusion in percent from three replicate RT-PCR experiments. Lines indicate relevant comparisons with asterisks representing different levels of significance (^∗∗∗^p < 10^−4^; n.s., not significant; Student’s t test). Error bars represent SDM. (D) Bar diagram depicting the fraction of downregulated genes within sets of genes carrying *Alu* exons with different levels of upregulation in KD1 (intervals of the fold change in inclusion in KD1 are given below). See also Table S5.

Erroneous *Alu* exon inclusion has been implicated in various diseases (Vorechovsky, 2010). For instance, exonization of an intronic *Alu* element in the *PTS* gene has been associated with hyperphenylalaninemia (Meili et al., 2009): deletion of the upstream U-tract leads to *Alu* exon inclusion from a downstream, cytidine-rich polypyrimidine tract. We observe strong hnRNP C binding at this U-tract, and exonization of the *Alu* element in the *HNRNPC* knockdown (Figure 6A; Data S1A). We designed a *PTS* minigene to test whether the disease mutation disrupts the ability of hnRNP C to repress this *Alu* exon (Figure 6A). As expected, there is almost no *Alu* exon inclusion in the control HeLa cells, while *HNRNPC* knockdown leads to a strong increase in aberrant exonization (Figures 6B and 6C). As previously described (Meili et al., 2009), introduction of the disease-associated mutation increases the aberrant exon inclusion in the control samples. Importantly, we show that hnRNP C depletion does not produce any additional effect (Figures 6B and 6C). This suggests that hnRNP-C-dependent repression is completely abolished by the clinically relevant mutation. These experiments demonstrate that hnRNP C binding is crucial for preventing the unwanted exonization of this *Alu* element under normal conditions.

We also assessed the broader protective function of hnRNP C in maintaining transcriptome integrity. Almost 80% of the *Alu* exons in our RNA-seq data are predicted to introduce frameshifts or stop codons that will strongly impair the function of the 1,572 genes containing them. Once included in processed transcripts, these *Alu* elements are likely to impair the function of the final protein product and could target the respective transcripts into the nonsense-mediated decay pathway (Mendell et al., 2004). In line with *Alu* exon-induced transcript degradation, we observe a correlation between *Alu* exon inclusion and downregulation of the corresponding transcripts in the *HNRNPC* knockdown (Figures 6D and S4F). hnRNP C’s importance is further underlined by the observation that the affected transcripts are implicated in a broad range of cellular functions (Table S4). For instance, hnRNP C represses *Alu* exons in *BAX, VHL, RAD52,* and *HELLS,* which encode proteins with key functions during development and disease.

## Discussion

A growing catalog of genome-wide CLIP studies continues to generate fascinating insights into the diverse functions of RNA-binding proteins. Combining these data with additional functional information allows us to interpret the consequences of RNA binding in diverse cellular processes, including alternative splicing, 3′ end processing, and translation (Darnell et al., 2011; Hafner et al., 2010; König et al., 2010, 2012; Licatalosi et al., 2008; Ule et al., 2003; Wang et al., 2012).

In this study, we developed the iCLIP approach a step further: the refined technique allowed us not only to discover many protein-RNA interactions, but also to quantify the relative strengths of these associations under different conditions. This enabled the quantitative measurement of competitive binding between two RNA-binding proteins on a transcriptome-wide scale. Specifically, our study combines experimental and computational genomic approaches to describe a general mechanism for regulating splicing via competitive RNA binding, uncover a safeguarding mechanism for transcriptome integrity, and provide insights into *Alu*-derived exon evolution.

### How Competitive Binding Determines the Splicing Outcome

Many splicing decisions are made in the early phases of spliceosome assembly (Wahl et al., 2009). An important checkpoint is the binding of U2AF65 to the polypyrimidine tract, which is targeted by multiple regulators (Wahl et al., 2009). U2AF65 has a broad binding specificity for motifs comprising both uridines and cytidines, leading to recognition of a very heterogeneous spectrum of polypyrimidine tracts (Singh et al., 2000). hnRNP C’s comparatively strict specificity for U-tracts allows hnRNP C to selectively compete with U2AF65 on a subset of sites, most prominently at cryptic splice sites within *Alu* elements (see below). U2AF65’s degenerate specificity also opens the possibility of competitive binding with other regulators at defined subsets of sites. Accordingly, many splicing factors apart from hnRNP C are recruited to specific sets of polypyrimidine tracts to regulate downstream exons (Licatalosi et al., 2008; Llorian et al., 2010; Wang et al., 2012; Xue et al., 2009), and some of these were shown to modulate U2AF65 binding. For instance, competitive binding by PTB to the *β-tropomyosin* transcript is associated with reduced U2AF65 binding and decreased exon 6 inclusion (Saulière et al., 2006). Similarly, hnRNP A/B proteins prevent U2AF65 binding to an alternative splice site in HIV-1 pre-mRNA by polymerizing across the polypyrimidine tract (Domsic et al., 2003). These observations underline the crucial role of the polypyrimidine tract as a regulatory hub, which enables the interplay of multiple regulators with the splicing machinery.

### hnRNP C Prevents Spurious U2AF65 Recognition of Cryptic Splice Sites

**Figure 7:**
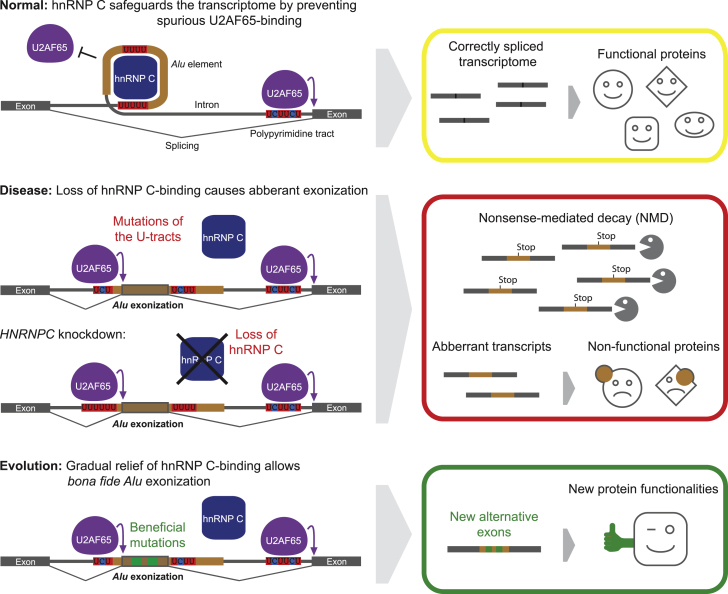
hnRNP C Safeguards the Transcriptome from the Exonization of *Alu* Elements In normal cells, hnRNP C prevents recognition of the *Alu* elements through U2AF65, thereby ensuring accurate splicing. In the *HNRNPC* knockdown, U2AF65 can bind to the U-tracts and promote *Alu* exonization. Similarly, disease-associated mutations in the U-tracts can favor *Alu* exonization in the presence of hnRNP C by impairing hnRNP-C-binding. The resulting nonfunction transcripts are likely either targeted by nonsense-mediated decay (NMD) or give rise to nonfunctional proteins. Once an exon acquires beneficial changes during evolution, similar mutations accumulate to relieve hnRNP C repression, opening the possibility for new protein functionalities.

Perhaps the most striking result of the study is that hnRNP C binds to more than 70,000 *Alu* elements, and that the absence of hnRNP C gives rise to more than a thousand cryptic *Alu* exons. hnRNP C prevents exonization of the *Alu* elements by strongly binding to their U-tracts. Indeed, our minigene experiments suggest that hnRNP C’s competition with U2AF65 at U-tracts upstream of 3′ splice sites constitutes a major mechanism of *Alu* exon repression (Figure 7). In addition to the effect at 3′ splice sites, we also detected hnRNP C binding to U-tracts downstream of 5′ splice sites. Here, hnRNP C might interfere with binding of TIA1 and TIAL, which were previously described to enhance 5′ splice-site usage in *Alu* exons (Förch et al., 2002; Gal-Mark et al., 2009). In addition, simultaneous binding to both U-tracts might aid the formation of stable hnRNP particles (Huang et al., 1994; König et al., 2010), which would in turn reinforce hnRNP C’s capacity to compete with U2AF65 and other splicing factors.

*Alu* exon repression is specific for hnRNP C. In particular, we could exclude an involvement of hnRNP A1, which was previously shown to proofread U2AF binding (Tavanez et al., 2012). This is not unexpected, because hnRNP A1 proofreading relies on the absence of an AG dinucleotide in the 3′ splice site, which is commonly present downstream of U-tracts in *Alu* elements. Similarly, depletion of other regulators like TIA1, TIAL, TDP-43, and PTB did not trigger the inclusion of cryptic *Alu* exons.

### Deleterious Consequences of Aberrant *Alu* Exonization in the Absence of hnRNP C

The increasing number of reported *Alu*-associated disorders illustrates that the enormous amounts of *Alu* elements pose a serious threat to the normal function of human cells (Hedges and Deininger, 2007; Kreahling and Graveley, 2004). Diseases like congenital cataracts facial dysmorphism neuropathy syndrome are caused by the inclusion of intronic *Alu* elements that severely disrupt the transcript structure, thereby affecting the function of the resulting protein (Varon et al., 2003). Indeed, a recent study estimated that 11 of 78 documented genetic diseases involving cryptic exons are associated with mutations in *Alu* elements (Vorechovsky, 2010). Although some studies have previously suggested the involvement of *trans*-acting factors (Lin et al., 2009; Shen et al., 2011; Sorek et al., 2002), the mechanisms by which cells protect against spurious exonization of *Alu* elements has remained unknown until now.

Our study establishes that one of hnRNP C’s principal functions is to protect the human transcriptome from aberrant *Alu* exonization. It is important to note that our current analysis of 2,000 *Alu* exons most likely underestimates the full scale of aberrant exonization in the absence of hnRNP C; this is because *Alu* elements are notoriously difficult to detect by current RNA-seq methods (Treangen and Salzberg, 2012). Furthermore, although the present study focuses on *Alu* elements because they represent the largest family of retrotransposons in the human genome, we suggest that hnRNP C might be important for suppressing retrotransposon-derived exons in general. Their poly(A) tails, and hence U-tracts when in antisense orientation, are required for efficient retrotransposition (Deininger, 2011; Dewannieux et al., 2003). In summary, we propose that hnRNP C plays a critical role in preserving human health by safeguarding transcriptome integrity against the detrimental effects of spurious exonization.

### hnRNP-C-Mediated Repression May Also Facilitate Evolutionary Innovation

More than 650,000 *Alu* elements reside within the transcribed regions of the human genome. Although we have stressed the threat posed by the loss of hnRNP C repression, many studies have highlighted the potential for *Alu* exonization to introduce genomic variation and evolutionary innovations (Häsler et al., 2007; Lev-Maor et al., 2003; Schmitz and Brosius, 2011; Shen et al., 2011). A well-studied example is the inclusion of the *Alu* exon in the *CD55* gene, which is regulated by hnRNP C and converts the encoded protein from a membrane-bound to a secreted version (Caras et al., 1987). Genuine *Alu*-derived exons are estimated to contribute 5% of all internal alternative exons: they are particularly enriched among recently acquired exons (Sela et al., 2007; Sorek, 2007; Vorechovsky, 2010) and are present in half of the human-specific genes, underlining their likely involvement in genome evolution and species-specific adaptation (Keren et al., 2010; Shen et al., 2011; Toll-Riera et al., 2009).

While the evolutionary potential of *Alu* exonization has attracted considerable interest (Sorek, 2007), the sudden incorporation of *Alu* elements into mature transcripts is likely to be deleterious in the vast majority of cases. In this context, the role of hnRNP C as a global suppressor may have important implications for evolutionary adaptation: in the presence of hnRNP C, *Alu* elements are repressed instead of being removed from the genome through selection, allowing them to evolve near-neutrally for longer evolutionary times. Cryptic *Alu* exons that are deleterious will remain suppressed; however if an exon becomes less deleterious by chance, selection against exonization will be considerably reduced. Mutations to the U-tracts that change the balance of binding between hnRNP C and splicing factors may allow low levels of “leaky” exonization, which allows even stronger evolutionary testing by selection. This could also be achieved by recruitment of additional factors that stabilize spliceosome binding and counteract hnRNP C interference, thereby circumventing the need to completely remove long U-tracts. Sequential mutations would thus enable an incremental exonization process, which could eventually lead to loss of hnRNP-C-dependent repression if an exon becomes functional and provides adaptive potential (Figure 7).

In conclusion, we propose that hnRNP C plays a critical role in protecting the transcriptome from the harmful effects of aberrant *Alu* exonization, while stabilizing a large reservoir of *Alu* elements in the human genome to facilitate the evolutionary exploration of new functions. The hnRNP-C-mediated regulation of *Alu* exonization has important implications for the evolution of the human genome and disease progression.

## Experimental Procedures

### RNA-Seq Analyses

RNA-seq libraries were sequenced on an Illumina GA-2 (72 cycles, paired end) and mapped to the human genome hg19 using TopHat (Trapnell et al., 2009; Table S1).

### iCLIP Experiments

iCLIP experiments were performed as described in König et al. (2011) using monoclonal mouse antibody (4F4) from Santa Cruz (sc-32308) for hnRNP C and a monoclonal mouse antibody (MC3) from Sigma (U4758) for U2AF65 (Tables S1 and S5). A summary of major steps can be found in the legends of Figures S2A and S2B.

### *HNRNPC* Knockdown

Knockdown of *HNRNPC* in HeLa cells was achieved with hnRNP C Stealth Select RNAi siRNAs HSS179304 and HSS179305 as well as control siRNA Stealth RNAi siRNA Negative Control (Invitrogen).

### Expression of Recombinant Proteins

Recombinant glutathione S transferase (GST)-tagged full-length hnRNP C1 and His-tagged U2AF65^RRM12^ comprising residues 148–342 were purified from *Escherichia coli* BL21-CodonPlus(DE3)-RP cells (Stratagene).

### De Novo Exon Prediction and Classification

Exon coordinates were predicted using Cufflinks (Trapnell et al., 2010) followed by several quality filters (Table S2). *Alu* exons were defined as exons with at least one splice site within an antisense *Alu* element (taken from RepBase; Jurka et al., 2005) that was supported by at least one junction-spanning read.

### Calculation of RBP Occupancy and Differential Binding

To correct RBP occupancy for changes in gene expression, we normalized each binding site to the total amount of crosslinking within the respective gene. Differential binding of U2AF65 was assessed using the log_2_-transformed ratio of normalized occupancies (KD/Ctrl). To allow direct comparison of U2AF65 binding from *HNRNPC* knockdown and control samples, we corrected the occupancies for the different iCLIP library sizes using DESeq (Anders and Huber, 2010).

Further experimental and computational methods are described in Extended Experimental Procedures.

Extended Experimental Procedures **Cell Culture**

HeLa cells were grown in Dulbecco’s modified Eagle medium (DMEM) supplemented with 10% Fetal Bovine Serum and 1% penicillin-streptomycin and cultured at 37°C with 5% CO_2_.

**Knockdown of HNRNPC and Minigene Reporter Transfections**

For the knockdown of *HNRNPC*, HeLa cells were plated at concentration of 10^5^ cells/well in a 6-well cluster plate in 2 ml of growth medium. 24 hr after the plating (when confluency reached around 20%), cultures were independently transfected using two different hnRNP C Stealth Select RNAi siRNAs (KD1 and KD2 refer to siRNAs HSS179304 and HSS179305 from Invitrogen, respectively) at a final concentration of 5 nM. Both siRNAs were transfected using Lipofectamine RNAiMAX (Invitrogen) according to the manufacturer’s instructions. Using the same procedure, we used Stealth RNAi siRNA Negative Control (Invitrogen) for the control experiments.

We performed Western analyses using hnRNP C-, U2AF65- and GAPDH-specific antibodies (monoclonal mouse hnRNP C antibody [4F4] from Santa Cruz, sc-32308; monoclonal mouse U2AF65 antibody [MC3] from Sigma, U4758; polyclonal rabbit U2AF65 antibody [H-300] from Santa Cruz Biotechnology, sc-48804; monoclonal rabbit GAPDH antibody [14c10] from Cell Signaling, 2118).

Following *HNRNPC* knockdown transfections, half an hour later cells were separately co-transfected with 400 ng of minigene plasmid using PolyFect reagent (QIAGEN), as recommended by the manufacturer. 48 hr after transfections, we collected cells and extracted total RNA using RNeasy Plus Mini kits (QIAGEN) for further analyses. All transfection experiments were performed in triplicates.

**Knockdown of other RNA-Binding Proteins**

The knockdown of *TDP-43* and the double knockdown of *TIA1/TIAL* in HeLa cells were performed as described earlier (Tollervey et al., 2011; Wang et al., 2010). RNA prepared from *PTB/nPTB* double knockdown and control HeLa cells according to Spellman et al., 2007 was a kind gift from Miguel Coelho and Chris Smith (University Cambridge). The *HNRNPA1* knockdown in HeLa cells was performed as described earlier (Tavanez et al., 2012) replacing Lipofectamine 2000 with Lipofectamin RNAimax (Invitrogen).

**iCLIP Experiments**

The method individual-nucleotide resolution UV-crosslinking and immunoprecipitation (iCLIP) allows the genome-wide identification of nucleotides that have been UV-crosslinked to the RNA-binding protein of interest (‘crosslink sites’). All iCLIP experiments were performed as described in König et al. (2011). A summary of major steps of the protocol can be found in the legend of Figures S2A and S2B.

For immunoprecipitation, we used a monoclonal mouse antibody (4F4) from Santa Cruz (sc-32308) for hnRNP C and a monoclonal mouse antibody (MC3) from Sigma (U4758) for U2AF65. In case of U2AF65, iCLIP experiments were performed 48 hr upon transfection with the hnRNP C-specific or control siRNAs (see above).

For hnRNP C, we obtained a total of 14 million unique crosslink events from two replicate iCLIP experiments from untreated HeLa cells (Table S1). For U2AF65, we performed two replicate iCLIP experiments from HeLa cells that were either treated with siRNA1 (KD1) or a mock siRNA (Ctrl) and one iCLIP replicate from HeLa cells treated with siRNA2 (KD2). These experiments identified a total of 27 million U2AF65 crosslink events, including 12 million crosslink events from control HeLa cells and 15 million from *HNRNPC* knockdown experiments (Table S1).

**Expression of Recombinant hnRNP C1 and U2AF65^RRM12^**

For expression of full-length hnRNP C1 as a GST fusion protein, a fragment encoding the full-length protein was amplified using PCR from IMAGE clone 6187512 (BC103758) and subcloned into a pGEX-6P-1 vector (GE Healthcare) using BamHI and XhoI restriction sites. The plasmid containing the open reading frame for the N-terminally His-tagged U2AF65 fragment comprising residues 148-342 (U2AF65^RRM12^) was a generous gift from the Sattler group (TU Muenchen, Germany; Mackereth et al., 2011).

Plasmid DNA was transformed into *E. coli* BL21-CodonPlus(DE3)-RP cells (Stratagene). Freshly transformed cells were cultured in LB medium, containing 30 μg/ml chloramphenicol and 100 μg/ml ampicillin (hnRNP C1 expression) or 30 μg/ml kanamycin (U2AF65 expression). Proteins were expressed over night at 20°C after induction with 0.25 mM IPTG. Protein purification was carried out at 4°C throughout.

For hnRNP C1 purification, harvested cells were resuspended in lysis buffer (50 mM Tris pH 7.4, 1 M NaCl, 12.5% (w/v) sucrose and 1 mM DTT) containing EDTA-free Complete Protease Inhibitor Cocktail (Roche) and lysed by sonication. The lysate was cleared by centrifugation and the supernatant incubated with Glutathione-Sepharose 4B (GE Healthcare), equilibrated in wash buffer (50 mM Tris pH 7.4, 1 M NaCl, 1 mM DTT and EDTA-free Complete Protease Inhibitor Cocktail). Protein-bound beads were washed thoroughly with wash buffer and equilibrated into cleavage buffer (50 mM Tris pH 7.4, 250 mM NaCl, and 1 mM DTT). The GST fusion protein was cleaved from the Glutathione-Sepharose beads by GST-tagged Precision Protease (GE Healthcare), leaving vector-derived residues Gly-Pro-Leu-Gly-Ser at the N-terminus of the protein.

U2AF65^RRM12^ was purified essentially as described in Mackereth et al. (2011). Harvested cells were resuspended in Ni-NTA binding buffer (50 mM Tris pH 7.4, 500 mM NaCl, 5% (v/v) Glycerol and 0.1% (v/v) Triton X-100) containing EDTA-free Complete Protease Inhibitor Cocktail (Roche) and lysed by sonication. The lysate was cleared by centrifugation and the protein was purified by using a 5 ml Ni-NTA HisTrap HP column (GE Healthcare) eluting with a linear imidazole gradient. The protein was equilibrated into 50 mM Tris pH 7.4, 500 mM NaCl, 5% (v/v) Glycerol and 1 mM DTT, and the N-terminal His-tag was cleaved by TEV protease, leaving vector-derived residues Gly-Ala-Met N-terminal of U2AF65 residues 148-342. Uncleaved protein, cleaved His-tag as well as His-tagged TEV protease were removed by repeating the Ni-NTA affinity purification and collecting cleaved U2AF65^RRM12^ in the flow-through. Purified hnRNP C1 and U2AF65^RRM12^ were concentrated using Vivaspin 2 columns (MWCO 3000Da, Sartorius).

**In Vitro UV Crosslinking Assays**

Binding reactions were carried out in binding buffer (10 mM Tris pH7.4; 100 mM KCl; 2.5 mM MgCl_2_) that we adopted from Görlach et al. (1994) replacing HEPES-KOH for Tris-Buffer. 10% of the RNA oligonucleotides (U_10_ and U_2_CU_4_CU_2_; Sigma) were radioactively labeled using PNK, and unincorporated γ-^32^P-ATP (Hartman) was removed using G-25 columns (GE Healthcare). RNA oligonucleotides were used at a final concentration of 100 nM, with protein concentrations as indicated in the figures. Binding reactions (20 μl) were incubated for 15 min at 30°C before the samples were UV crosslinked with 150 mJ/cm^2^ in a Stratalinker 2400 at 254 nm. Finally, the samples were analyzed on NuPAGE Bis-Tris gels (4%–12%; MES running buffer; Invitrogen) that were first exposed to an autoradiography film (Fujifilm) and then stained with Coomassie to control protein loading.

**RNA-Seq Analyses**

We performed RNA-seq experiments on two replicate samples from each *HNRNPC* knockdown (KD1 and KD2) as well as from control HeLa cells. Library preparation was preformed according to the mRNA Sequencing Sample Preparation Guide (Illumina, part # 1004898 REV. D). Reagents were taken from the Illumina sample preparation kit (Illumina, CAT # RS-930-1001). Knockdown and control samples were sequenced together in one flowcell on one and two lanes, respectively. The reads from the two lanes of each control samples were combined for all analyses.

**High-Throughput Sequencing and Genomic Mapping**

High-throughput sequencing of iCLIP and RNA-seq cDNA libraries was performed on an Illumina GA-2 (run length 54 nt for iCLIP and 72 nt for RNA-seq). The iCLIP libraries contained a 4-nt experimental barcode plus a 5-nt random barcode, which allowed multiplexing and the removal of PCR duplicates, respectively (Table S5). We obtained a total of 240 million 72-nt paired-end sequence reads for RNA-seq and 77 million reads for iCLIP (Table S1). All genomic analyses were performed using the human genome version hg19/NCBI37 with annotations taken from Ensembl (version 60; Flicek et al., 2011). The RNA-seq data are available from ArrayExpress (http://www.ebi.ac.uk/arrayexpress/) under the accession number E-MTAB-1147.

The iCLIP data were mapped using Bowtie (Langmead et al., 2009) and further processed as described previously (König et al., 2010). It is important to note that all iCLIP data sets showed comparable qualities and were mapped with the same parameters, since any mapping inconsistencies could lead to artifactual increases or decreases in the observed binding frequencies. Raw read sequences as well as processed data files are available from ArrayExpress (http://www.ebi.ac.uk/arrayexpress/) under the accession number E-MTAB-1371.

For assessing the genomic distribution of iCLIP crosslink nucleotides, we used the following hierarchy: ncRNA > 3′ UTR > 5′ UTR > exon > intron > antisense > intergenic (Figure S1A). Introns were further subdivided into the regions 200 nucleotides before and after exons and “deep-intronic” positions more than 200 nucleotides away from any annotated exon.

The RNA-seq data were mapped using the splice-aware alignment algorithm TopHat version 1.1.4 (Trapnell et al., 2009) based on the following parameters: tophat -num-threads 8 -mate-inner-dist 200 -solexa-quals -min-isoform-fraction 0 -coverage-search -segment-mismatches 1. More than 80% of all RNA-seq reads mapped uniquely to the human genome (Table S1), and 83% of these mapped as part of perfect read pairs.

**De Novo Exon Prediction and Classification**

In order to predict exons from our RNA-seq data, we ran Cufflinks (version 0.9.3, -min-isoform-fraction 0 to allow detection of weakly included exons; Trapnell et al., 2010) on the collapsed reads from all control and *HNRNPC* knockdown samples and then extracted the exons of all predicted transcripts. In order to minimize noise due to erroneous or highly overlapping annotations, we kept only exons that (a) were predicted as part of multi-exon transcripts, (b) were supported by at least one junction-spanning read, and (c) had a size of at least 25 bp and no more than 10 kb. Finally, overlapping exons that started/ended less than 25 bp from each other were merged into one exon using the outer exon boundaries. Applying these filters, Cufflinks predicted a total of 178,029 exons (reported as ‘Total’ in Table S2), including 16,143 exons that did not overlap with any exon in the Ensembl database and thus represented good candidates for cryptic exons. We used Ensembl gene annotations to assign the exons to gene models. Exons that directly overlapped with an Ensembl gene or that were part of a predicted Cufflinks gene model that overlapped with only one Ensembl gene were assigned to that gene. We discarded exons that overlapped with more than one Ensembl gene. Applying this procedure, Cufflinks-predicted exons could be associated with 14,091 Ensembl genes. For the alternative splicing analyses using DEXSeq, we further restricted the set to exons that did not overlap with any other annotated exon (“Non-overlapping” in Table S2).

To identify *Alu* exons, we searched for Cufflinks-predicted exons that contained at least one splice site within an antisense *Alu* element (taken from RepBase; see below). The *Alu*-derived splice site had to be supported by at least one junction-spanning read in our collapsed RNA-seq data. Following this definition, Cufflinks exon predicted a total 2,085 *Alu* exons (Table S2). 1,376 of these were predicted to introduce a frameshift, and 472, 610, and 797 harbored a stop codon in one, two and all three frames, respectively. Combining this evidence, 1,655 (79%) of the *Alu* exons were predicted to disrupt the respective transcript upon inclusion. 1,903 of the *Alu* exons did not overlap with any other exon prediction (nonoverlapping) and were thus used for all following analyses, including 1,318 *Alu* exons that did not overlap with any Ensembl annotation. We notice that some *Alu* exons are expressed at negligible levels in the control sample; however given that they remain unannotated and they are largely undetectable in our RNA-seq data, they are unlikely to play an important role under normal conditions. We further identified a control set of 81 established *Alu* exons that show substantial inclusion already in control HeLa cells and that do no longer underlie hnRNP C regulation (*Alu* exons harboring a total of at least 50 reads in our RNA-seq samples and showing a fold change < 1.5 [KD/ctrl]).

**Gene Ontology Analysis of Alu Exon-Containing Genes**

We used the PANTHER (Protein ANalysis THrough Evolutionary Relationships) Classification System (Thomas et al., 2003) to identify the GO Biological Processes associated with the 1,572 genes containing the *Alu* exons detected in our RNA-seq analyses (Table S4).

**Alignments of Alu Elements**

We used *Alu* element annotations based on RepeatMasker predictions (Smit et al., 1996–2010) taken from RepBase (Jurka et al., 2005). Based on Ensembl gene annotations, the total of 1,194,727 predicted *Alu* elements were classified into ‘sense’ (located on the same strand as the overlapping gene; 282,706), ‘antisense’ (located on the opposite strand to the overlapping gene; 342,016), ‘ambiguous’ (overlapping with genes on both strands; 35,216) and ‘intergenic’ (not overlapping with any annotated gene; 534,789). Assessing hnRNP C binding to the *Alu* elements, we found that 72,625 of *Alu* elements in antisense orientation (21%) and 79 of *Alu* elements in sense orientation (0.03%) overlapped with an hnRNP-C-binding site (3,957/11% of ambiguous and 10,187/2% of intergenic).

Our RNA-seq data show exonization (see below) of 1,875 *Alu* elements in antisense orientation (compared to 182 *Alu* elements in sense orientation that harbor the splice site of an exon). In order to map the splice-site positions back to the *Alu* consensus sequence, we used PRANK-F (with default parameters for noncoding DNA sequences; Löytynoja and Goldman, 2008) to align the predicted *Alu* elements to the consensus sequence taken from RepBase (Jurka et al., 2005). To maximize the reliability of the alignments and to simplify the interpretation of the results, we restricted the analysis to full-length *Alu* elements in the human genome. In particular, we aligned the canonical *Alu* sequence to the 856,791 *Alu* elements in the human genome whose length was within ± 15% of the length of the canonical *Alu* consensus sequence. To improve the quality of the alignments we used multiple sequence alignment instead of pairwise alignment, aligning the sequences in 21,969 groups of 40 sequences. Based on the alignments, each genomic position within the *Alu* element was assigned to a nucleotide position within the consensus sequence. This mapping was then used to assess the position of *Alu* exons, the usage of 3′ and 5′ splice sites (Figures 3F, S4D, and S4E; only exons where both splice sites are supported by junction-spanning reads were taken into account) and the binding of hnRNP C on the *Alu* consensus (Figure 3F).

**Evolutionary Analyses**

The detection of selection signatures in this context is challenging for several reasons. First, even if *Alu* elements are likely to evolve almost neutrally, the evolutionary divergence among paralogous copies is limited given their relatively recent expansion. Second, any analysis should consider that the insertion/deletion rate of U-tracts is likely to be much higher than the mutation rate of more complex flanking sequences, owing to processes such as polymerase slippage (Leclercq et al., 2010). Third, different types of mutations (point mutations, insertions and deletions) may lead to analogous changes in the length of continuous U-tracts, and so they may have similar phenotypic consequences. Thus, to account for these limitations, we compared the frequency of unusually short and unusually long U-tracts in hnRNP C-regulated *Alu* elements with these frequencies in a matched set of nonexonized control exons (nonexonized intronic *Alu* elements from the same transcripts as the regulated set).

We restricted this analysis to *Alu* exons arising from the most commonly used 3′ splice sites (corresponding to the enlarged sequence windows shown in Figure S4E) and further separated them according to the location of the 3′ splice site in the first or second arm of the *Alu* element. This yielded 956 and 333 *Alu* elements with exonization from the first and second arm, respectively. Figure 3G depicts the ratio between: (a) the fraction of U-tracts longer than a given length in each set and (b) the corresponding fraction in its own reference set of nonexonized antisense *Alu* elements within the same genes (29,712 and 11,509 *Alu* elements, respectively). Significance was assessed using Pearson’s chi-square test (or Fisher’s exact test when one or more values in the contingency table are below five). As a control, we also show these ratios for nonexonized *Alu* elements of all other genes (gray line).

**Analyses of Differential Gene Expression/Splicing**

Analyses of differential gene expression were performed using DESeq (Anders and Huber, 2010) based on Ensembl gene annotations (version 60; Flicek et al., 2011). We identified 4,880 and 4,875 genes that showed significant differential expression in KD1 and KD2, respectively (adjusted p value < 0.01). Since the obtained fold change values showed strong correlation between both knockdowns (r = 0.635, Pearson’s product-moment correlation), we combined the results of both knockdowns using the conditional thresholding approach described below. This yielded a high-confidence set of 4,636 genes showing differential regulation, including 1,981 upregulated and 2,655 downregulated genes.

Differential splicing was determined using DEXSeq (Anders et al., 2012) based on Cufflinks-predicted, nonoverlapping exons, where reads could be assigned unambiguously. This approach identified 3,452 and 1,197 significantly differentially spliced exons in KD1 and KD2, respectively (adjusted p value < 0.05). Since detected splicing changes were strongly correlated between both knockdowns (r = 0.57, Pearson’s product-moment correlation; Figure S4A), we combined the results from both knockdowns using the conditional thresholding approach described below. This identified a high-confidence set of 3,052 exons that were differentially regulated in both knockdowns, including 1,807 upregulated and 1,245 downregulated exons (Figure S4B and Table S2). We refer to these exons as being repressed and enhanced by hnRNP C, respectively.

For both methods, we used information from all three sample types (Ctrl, KD1 and KD2) to determine the variance between biological replicates which was then used in the individual comparisons (e.g., Ctrl versus KD1). In order to integrate the data obtained from the two different *HNRNPC* knockdowns (KD1 and KD2), we conceived a conditional thresholding approach that was applied to assess differential gene expression (based on DESeq results) as well as differential exon usage (based on DEXSeq results). To obtain a collapsed set of differentially expressed genes/exons, we required each gene/exon to obtain a p value < 0.01 in at least one of the knockdowns and at least p value < 0.05 in the second knockdown.

**Characterization of RBP-Binding Sites**

In order to identify hnRNP C binding sites, we applied a peak-finding algorithm that identified clusters of crosslink nucleotides with significant enrichment of crosslink events relative to the local environment (König et al., 2010). For hnRNP C, we used a flank size of 10 nt to either side to define significant clusters with FDR < 5% on the combined replicates from untreated HeLa cells. This yielded a total of 438,360 binding sites, 78% of which were supported by both replicates. In order to assess hnRNP C binding to U-tracts, we obtained the coordinates of all continuous tracts of three or more uridines in the transcriptome and determined the length of the longest overlapping tract for each binding site.

In order to identify U2AF65-binding sites, we used a flank size of 5 nt with FDR < 5%, since U2AF65 shows much more focused binding than hnRNP C. The cluster definition was performed on the collapsed data from the control and both *HNRNPC* knockdown experiments, yielding a total of 518,794 significant binding sites (Table S1). 59% of these binding sites were supported by both replicates from control HeLa cells, and the number of crosslink events within the binding site was highly correlated between the replicates (Figure S3A).

Overlaying U2AF65 binding with Ensembl exon annotations, we found U2AF65-binding sites in the last 50 nt upstream of 35% of all 3′ splice sites (76,055 out of 219,864 unique 3′ splice sites of all Ensembl-annotated exons), corresponding to 58% of all 3′ splice sites that we detected in HeLa cells under the studied conditions (71,918 out of 123,063 3′ splice sites that are supported by at least two junction-spanning reads in our collapsed RNA-seq data), underlining the prominent role of U2AF65 in 3′ splice-site recognition.

**Calculation of RBP Occupancy and Differential Binding**

To correct for changes in gene expression, we normalized the number of crosslink events within each cluster to the total amount of crosslinking on the respective gene. Based on the significant binding sites that we obtained from our FDR approach, we first counted the number of crosslink events within each binding site for each sample type. The raw counts of the biological replicates were used to assess the reproducibility between replicates (Figure S3A) as well as between the two different *HNRNPC* knockdowns (Figure S3B) and then summed up. Since the measured occupancy depends not only on the affinity of the RBP for a given site, but also on the expression level of the whole transcript, we normalized the crosslink events within the binding sites to the total count within the respective gene (based on Ensembl gene annotations). To this end, we filtered for binding sites that located within and did not overlap with more than one annotated gene. We then obtained a total gene-wide count as the sum of crosslink events in binding sites within the gene boundaries. Finally, we calculated the normalized occupancy by dividing the sum of crosslink events within each binding site by the total count of the respective gene and the gene length. Since hnRNP C shows very broad widespread binding, we restricted the occupancy calculation to a 11-nt window centered around the nucleotide with most crosslink events within each binding site.

In order to assess differential binding of U2AF65 upon *HNRNPC* knockdown, we used the log2-transformed ratio of normalized occupancies (KD / Ctrl). Since the detected changes were highly correlated between both *HNRNPC* knockdowns (r = 0.545, Pearson’s product-moment correlation; Figure S3B), we combined the data sets generated from both knockdowns for the remaining analyses (referred to as KD).

Using a stringent fold change threshold, we identified 3,353 and 2,147 binding sites with at least four-fold increased or decreased U2AF65 binding upon *HNRNPC* knockdown, respectively. We defined a control set of 110,515 unchanged U2AF65-binding sites (log2(fold change KD / Ctrl) | < 0.1). We found overlapping hnRNP-C-binding sites for 1,698 (51%), 90 (4.2%) and 8,004 (7.2%) of U2AF65 binding sites with at least four-fold increased or decreased or unchanged U2AF65 occupancy in the *HNRNPC* knockdown.

To allow comparison of U2AF65 binding from *HNRNPC* knockdown and control samples, we corrected the occupancies for the different iCLIP library sizes. To achieve this, we used DESeq (Anders and Huber, 2010) to calculate the size factors of both conditions based on the occupancy values of all U2AF65-binding sites. We then applied to size factor to correct the values from the *HNRNPC* knockdown as they are shown in Figures 2I, 4, and S5D.

**Analyses of Publicly Available Binding Data for Other RBPs**

We examined the publicly available data on binding sites of ten other RNA-binding proteins, including CLIP-seq data for hnRNP A1 (1,956 binding sites), A2B1 (10,193), F (18,950), H (33,201), M (4,437), U (17,174; all data from HEK293 cells, Huelga et al., 2012) and PTB (51,389; data from HeLa cells, Xue et al., 2009) as well as iCLIP data for TIA1 (39,545), TIAL (95,516; all data from HeLa cells, Wang et al., 2010) and TDP-43 (95,999; data from hES cells, Tollervey et al., 2011).

To address the involvement of other RBPs in the hnRNP C-U2AF65 competition, we searched for overlapping RBP-binding sites within a window of 100 nt on either side of the summit of U2AF65-binding sites that overlap with hnRNP C. To allow direct comparison in Figure 2C, we normalized the frequency of overlapping clusters of a given RBP to the mean background frequency of the same RBP in two 10-nt windows 90 nucleotides up- and downstream of the U2AF65 summit position.

**Construction of Minigene Plasmids and Site-Directed Mutagenesis**

As template for minigene constructions and site-directed mutagenesis, we used PCR to amplify the respective regions from genomic DNA using Phusion High-Fidelity DNA Polymerase (NEB) to avoid the introduction of undesired point mutations. Sequencing of all constructs verified that control and mutated minigene plasmids were identical except for the mutated sites. All minigene constructs are based on the expression vector pcDNA3 (Invitrogen).

For the *CD55* wild-type reporter minigene (CD55_wt), we reduced the size of the intronic region downstream of the *Alu* element by two subsequent PCR steps using the oligonucleotides CD55_wt_F1 with CD55_wt_R1 and CD55_wt_F2 with CD55_wt_R2 oligonucleotides (see Table S5 for oligonucleotide sequences). The product was cut with NotI and XbaI and ligated into pcDNA3 opened with the same restriction enzymes. For the mutated *CD55* minigenes, specific mutations in the U-tracts of the *Alu* element were introduced through PCR on CD55_wt with the following oligonucleotides: CD55_3mut_R with CD55_wt_F1 and CD55_3mut_F with CD55_wt_R2 for mutating the upstream U-tract; CD55_5mut_R with CD55_wt_F1 and CD55_5mut_F with CD55_wt_R2 for mutating the linker U-tract. After a final PCR with the outer oligonucleotides, the products were ligated as NotI/XbaI fragments into pcDNA3, resulting in plasmids CD55_3mut and CD55_5mut, respectively. The *CD55* minigene combining mutations in both U-tracts was created by digesting both mutated plasmids with PstI and NotI, gel-purifying the fragments containing the mutated U-tract and then ligating these together.

For the *NUP133* wild-type reporter minigene (NUP133_wt), the respective region was amplified from human genomic DNA using oligonucleotides NUP133_wt_F and NUP133_wt_R. The PCR product was cut with BamHI and EcoRI and ligated into pcDNA3 opened with the same restriction enzymes. Mutations in the upstream U-tract of the *Alu* element (NUP133_3mut) were introduced through PCR with the oligonucleotides NUP133_wt_F with NUP133_3mut_R and NUP133_wt_R with NUP133_3mut_F on SmaI-linearized NUP133_wt plasmid DNA. After a third PCR with the outer oligonucleotides, the product was cut with BamHI and EcoRI and inserted into pcDNA3. Using the same approach, the plasmid NUP133_5mut with mutations in the linker U-tract was created using the oligonucleotides NUP133_5mut_F and NUP133_5mut_R. To combine both U-tract mutations into NUP133_3mut+5mut, we introduced the mutations into the linker U-tract into NUP133_3mut using NUP133_5mut_F and NUP133_5mut_R combined with the corresponding outer oligonucleotides. The product was cut using BamHI and EcoRI and ligated into pcDNA3.

For the *PTS* wild-type reporter minigene (PTS_wt), the respective region was amplified from human genomic DNA using oligonucleotides PTS_wt_F and PTS_wt_R. The product was cut with NotI and XbaI and ligated into pcDNA3. For the mutated minigene (PTS_disease), the disease-associated deletion was introduced by PCR on PTS_wt using oligonucleotides PTS_disease_F and PTS_disease_R. After the final PCR using the outer oligonucleotides, the product was cut with NotI and XbaI and ligated into pcDNA3.

**RT-PCR Reporter Minigene Assays**

We used quantitative RT-PCR to measure exon inclusion levels from the minigene constructs in control and *HNRNPC* knockdown HeLa cells (Figures 5, 6, and S6). For visualization of the PCR products and quantification of individual splicing isoforms, we used a QIAxcel capillary gel electrophoresis system (QIAGEN). Following RNA extraction, 500 ng of total RNA was reverse transcribed using the RevertAid Premium First Strand cDNA Synthesis Kit (Fermentas).

For the *PTS* minigenes (Figure 6), quantitative RT-PCR amplification was performed using IMMOLASE DNA Polymerase (Bioline) under the following conditions: 95°C for 10 min, 35 cycles of [95°C for 10 s, 55°C for 10 s, 72°C for 30 s], then finally 72°C for 2 min. In order to amplify only cDNAs derived from the *PTS* minigenes, we combined a vector-specific with a *PTS*-specific oligonucleotide (Vector_F with PTS_R, respectively; Table S5). For the *CD55* minigenes (Figure 5), we combined the same vector-specific oligonucleotide with the *CD55*-specific oligonucleotide CD55_R (Table S5) and performed 40 PCR cycles (as described above) to increase signal intensity.

Due to the larger PCR product sizes from the *NUP133* minigenes (Figure S6), we used Phusion High-Fidelity DNA Polymerase (NEB) with the vector-specific and the NUP133-specific oligonucleotides Vector_R and NUP133_F, respectively (Table S5), under the following amplification conditions: 98°C for 10 s, 32 cycles of [98°C for 1 s, 55°C for 5 s, 72°C for 30 s], and then 72°C for an additional minute. The PCR products were desalted by ethanol precipitation before quantification.

**RT-PCR Quantification of Endogenous Alu Exons and Control Exons**

In order to validate the splicing changes of *Alu* exons identified from our RNA-seq data (Data S1 and Table S3), total RNA extracted from HeLa cells was reverse transcribed using the RevertAid Premium First Strand cDNA Synthesis Kit (Fermentas). We then performed quantitative RT-PCR measurements using IMMOLASE DNA Polymerase (Bioline) under following conditions: 95°C for 10 min, 35 cycles of [95°C for 10 s, 55°C for 10 s, 72°C for 30 s] and then finally 72°C for 2 min. For each *Alu* exon, we used oligonucleotides annealing to both neighboring constitutive exons (Table S3). A QIAxcel capillary gel electrophoresis system was used to visualize the PCR products and quantify each isoform. All measurements were performed in triplicates. We could validate significant splicing changes in the *HNRNPC* knockdown in 55 out of the 63 (87%) tested *Alu* exons. Among these, we confirmed significant hnRNP C regulation of 16 out of 20 tested *Alu* exons that were not called by DEXSeq (Data S1).

To investigate the involvement of other RBPs in *Alu* exon repression, we investigated the inclusion of four cryptic *Alu* exons (*HELLS*, *KIAA1432*, *MBD3* and *PEX14*) and four Ensembl-annotated *Alu* exons (*CD55*, *MTO1*, *ZFX* and *WRN*; Table S3 and Figure 3E) in RBP knockdown and control samples as described above. To verify efficient knockdown, we also quantified the inclusion of one known target exons for each RBP: *PPP3CB* for hnRNP A1 (Venables et al., 2008), *MRPS18C* for TIA/TIAL (Wang et al., 2010), *POLDIP3* for TDP-43 (Tollervey et al., 2011) and *ANXA7* for PTB (Spellman et al., 2007; Table S3 and Data S1C).

## Accession Numbers

The ArrayExpress accession numbers for the RNA-seq and iCLIP data are E-MTAB-1147 and E-MTAB-1371, respectively.

## Supplemental Information

Document S1. Tables S1 and S2

Table S3. Validation of *Alu* Exon Changes Using RT-PCR, Related to Figure 3 and Data S1 (A and B) Validation of *Alu* exons that were called as differentially regulated using DEXSeq and conditional thresholding (A, Data S1A) or that are more than 2-fold upregulated in the RNA-seq data (B, Data S1B). The percent PCR change in each *HNRNPC* knockdown was calculated as the difference of average percent inclusion in knockdown minus wt (Figure 3C). Information on both validated and nonvalidated exons are given. (C) Primer sequences for RT-PCR quantifications of known target exons of TDP-43, TIA/TIAL, PTB/nPTB, and hnRNP A1 (Data S1C).

Table S4. Gene Ontology Analysis of the 1,572 Genes Containing *Alu* Exons, Related to Extended Experimental Procedures Gene Ontology Biological Processes identified using the PANTHER (Protein ANalysis THrough Evolutionary Relationships) Classification System. All categories with at least ten members are listed. See also “Gene Ontology Analysis of *Alu* Exon-Containing Genes” in Extended Experimental Procedures.

Table S5. Information on Oligonucleotides, Related to Figures 5, 6, and S6 (A) Oligonucleotides used for reverse transcription (RT) in iCLIP experiments. RT primers were phosphorylated at the 5′ end. Barcode sequences are given for each replicate experiment, consisting of the replicate-specific barcode (nucleotides 4–7) and the random barcode indicated by N for any nucleotide. (B) Oligonucleotides used for cloning of minigene reporter plasmids (see Extended Experimental Procedures). (C) Oligonucleotides used for RT-PCR quantifications of the minigene reporter constructs (Figures 5, 6 and S6).

Data S1. RT-PCR Analyses of RNA-Seq-Predicted Changes in *Alu* Exon Inclusion, Related to Figure 3 and Table S3 (A) We could validate 39 out of 43 tested *Alu* exons that show increased inclusion in the *HNRNPC* knockdown according to our DEXSeq analysis. Validations were performed by reverse transcription (RT)-PCR triplicate experiments from *HNRNPC* knockdown (KD1 and KD2) and control (Ctrl) HeLa cells. The gel view of capillary electrophoresis of the PCR products (left) and the signal quantification (bar diagram on the right) are shown for each validated exon. The sizes of the quantified fragments including (in) or excluding (ex) the *Alu* exon are marked on the right of the gel image (Table S3). Bar diagrams on the right depict the mean inclusion level under each condition. Error bars represent the standard deviation of the mean from three replicate experiments. The significance level (Student’s t test) of the detected splicing changes relative to control is indicated above the bars as follows: n.s., nonsignificant; ^∗^ p value < 0.05; ^∗∗^ p value < 0.001; ^∗∗∗^ p value < 0.0001. (B) We could validate 16 out of 20 tested *Alu* exons that show a more than two-fold increase in inclusion in the RNA-seq data of at least one of the *HNRNPC* knockdowns (Table S3). Validations were performed and presented as in (A). (C) Control RT-PCR experiments analyzing splicing changes of exons that are known to be regulated by TIA1/TIAL, PTB, hnRNP A1 and TDP-43 (see Table S3C). Experiments were performed in triplicates using the two *HNRNPC* knockdowns (KD1 and KD2), a *TIA1/TIAL* double knockdown, a *PTB/nPTB* double knockdown, individual knockdowns for *HNRNPA1* and *TDP-43* (all labeled as KD with the respective gene(s) indicated above) as well as control (Ctrl) HeLa cells. The gel view of capillary electrophoresis of the PCR products (right) and the signal quantification (bar diagram on the left) are shown for each exon. The sizes of the quantified fragments including (in) or excluding (ex) the regulated alternative exon are marked on the right of the gel image (Table S3). Bar diagrams on the right depict the mean inclusion (gray) and exclusion (white) level under each condition. Error bars represent the standard deviation of the mean from three replicate experiments. The significance level (Student’s t test) of the detected splicing changes relative to control is indicated as in (A).

Document S2. Article plus Supplemental Information
