# Supplementary material for: Direct Competition between hnRNP C and U2AF65 Protects the Transcriptome from the Exonization of Alu Elements
Source: Cell. 2013 Jan 31;152(3):453–66. doi: 10.1016/j.cell.2012.12.023 (PMC3629564; doi:10.1016/j.cell.2012.12.023)
Supplement: Document S1. Tables S1 and S2 [file mmc1.pdf]

Table S1. iCLIP and RNA-Seq Mapping Statistics, Related to Experimental Procedures

hnRNP C iCLIP	Replicate 1 (LUjh03[1])	Replicate 2 (LUjh25)	Total
Total reads	17,016,384	20,525,828	**37,542,212**
Total crosslink events	4,869,687	9,488,133	**14,357,820**
Total crosslink sites	4,412,114	8,131,878	**11,899,326**
Binding sites (Flank 10; FDR 0.05)	ND	ND	**438,360**
Binding sites in annotated genes	ND	ND	**392,818**

U2AF65 iCLIP (Ctrl)	Replicate 1 (LUjh23_1)	Replicate 2 (LUjh23_2)	Total
Total reads	2,478,269	15,555,645	**18,033,914**
Total crosslink events	1,781,117	10,135,903	**11,917,020**
Total crosslink sites	1,488,737	7,209,165	**8,214,397**
Binding sites (Flank 5; FDR 0.05)	ND	ND	**518,794**

U2AF65 iCLIP (KD1)	Replicate 1 (LUjh21_1)	Replicate 2 (LUjh21_2)	Total
Total reads	4,378,005	6,228,475	**10,606,480**
Total crosslink events	3,077,838	4,384,385	**7,462,223**
Total crosslink sites	2,626,609	3,596,131	**5,778,770**
Binding sites (Flank 5; FDR 0.05)	ND	ND	**330,674**

U2AF65 iCLIP (KD2)	Replicate 1 (LUjh22_1)		Total U2AF65
Total reads	11,330,534		**39,970,928**
Total crosslink events	8,227,587		**27,606,830**
Total crosslink sites	6,254,382		**16,994,031**
Binding sites (Flank 5; FDR 0.05)	363,844		**1,122,142**
Binding sites in annotated genes	ND		**996,784**

RNA-seq (Ctrl)	Replicate 1	Replicate 2	Total
Total reads	120,269,114	127,222,962	**247,492,076**
Mapped	102,679,885	101,888,778	**204,568,663**
Mapped (% of total reads)	85.4	80.1	**82.7**
Uniquely mapped	99,206,481	98,420,834	**197,627,315**
Uniquely mapped (% of mapped)	96.6	96.6	**96.6**
Junction-spanning	20,687,792	20,210,793	**40,898,585**

RNA-seq (KD1)	Replicate 1	Replicate 2	Total
Total reads	59,483,704	65,331,134	**124,814,838**
Mapped	51,049,794	55,786,169	**106,835,963**
Mapped (% of total reads)	85.8	85.4	**85.6**
Uniquely mapped	49,387,650	53,933,424	**103,321,074**
Uniquely mapped (% of mapped)	96.7	96.7	**96.7**
Junction-spanning	9,046,955	9,221,022	**18,267,977**

RNA-seq (KD2)	Replicate 1	Replicate 2	Total
Total reads	63,752,362	64,691,484	**128,443,846**
Mapped	55,201,140	54,369,665	**109,570,805**
Mapped (% of total reads)	86.6	84	**85.3**
Uniquely mapped	53,281,319	52,506,281	**105,787,600**
Uniquely mapped (% of mapped)	96.5	96.6	**96.5**
Junction-spanning	9,755,630	10,275,003	**20,030,633**

[1] Names in brackets indicate internally used library identifiers

For the iCLIP experiments, the number of total reads as well as of detected crosslink events and crosslink sites after mapping and random barcode evaluation are given for each replicate. In addition, binding sites were called on the collapsed iCLIP data of each sample type. U2AF65 iCLIP as well as RNA-seq experiments were performed from *HNRNPC* knockdown (KD1 and KD2) and control (Ctrl) HeLa cells. Summarized counts for the collapsed U2AF65 iCLIP data from both *HNRNPC* knockdowns and control HeLa cells ('Total U2AF65') can be found after U2AF65 iCLIP (KD2).

Table S2. Exon Predictions and Regulation, Related to Experimental Procedures

Exon predictions

**Category**	Total	Non-overlapping
Total Cufflinks exons	178,029	140,621
Corresponding Ensembl genes	14,091	13,975
Novel exons	16,143	13,875
Alu exons	2,085	1,903
Novel *Alu* exons	1,406	1,318

Exon regulation

**Conditional thresholding** (p value$_a$[1] < 0.01 & p value$_b$ < 0.05)	Total	**Up**	**Down**
Total exons	3,052	**1,807**	**1,245**
Novel exons	894	**754**	**140**
	29%	**41%**	**11%**
Alu exons	363	**361**	**2**

***Alu* exon regulation**	Total	**Up**	**Down**
Called by DEXSeq	363	**361**	**2**
>2-fold change, not called by DEXSeq	778	**662**	**116**
Total regulated	1,141	**1,023**	**118**

[1] For the conditional thresholding, the p value of differential exon inclusion has to be < 0.01 in at least one *HNRNPC* knockdown (p value$_a$) and < 0.05 in the second knockdown (p value$_b$).

Top: 'Total' refers to all exons that were predicted by Cufflinks (see Extended Experimental Procedures for additional quality filtering steps). 'Non-overlapping' refers to the restricted set of exons that did not overlap with any other annotated exon and that was used in the DEXSeq analyses. 'Novel' refers to exons that do not overlap with any exon in the Ensembl database (version 60). 'Alu exons' show at least one splice site within an antisense *Alu* element (taken from RepBase) that is supported by at least one junction-spanning read from the collapsed RNA-seq data. Bottom: 'Total' refers to the total number of differentially spliced exons (only stand-alone exons were taken into account). For the 'conditional thresholding', the p value of differential exon inclusion has to be < 0.01 in at least one *HNRNPC* knockdown (p value$_a$) and < 0.05 in the second knockdown (p value$_b$). In addition to the DEXSeq calls, we also report *Alu* exons that show an at least 2-fold change in inclusion.
