# Supplementary material for: Direct Competition between hnRNP C and U2AF65 Protects the Transcriptome from the Exonization of Alu Elements
Source: Cell. 2013 Jan 31;152(3):453–66. doi: 10.1016/j.cell.2012.12.023 (PMC3629564; doi:10.1016/j.cell.2012.12.023)
Supplement: Data S1. RT-PCR Analyses of RNA-Seq-Predicted Changes in Alu Exon Inclusion, Related to Figure 3 and Table S3 — (A) We could validate 39 out of 43 tested Alu exons that show increased inclusion in the HNRNPC knockdown according to our DEXSeq analysis. Validations were performed by reverse transcription (RT)-PCR triplicate experiments from HNRNPC knockdown (KD1 and KD2) and control (Ctrl) HeLa cells. The gel view of capillary electrophoresis of the PCR products (left) and the signal quantification (bar diagram on the right) are shown for each validated exon. The sizes of the quantified fragments including (in) or excluding (ex) the Alu exon are marked on the right of the gel image (Table S3). Bar diagrams on the right depict the mean inclusion level under each condition. Error bars represent the standard deviation of the mean from three replicate experiments. The significance level (Student’s t test) of the detected splicing changes relative to control is indicated above the bars as follows: n.s., nonsignificant; ∗ p value < 0.05; ∗∗ p value < 0.001; ∗∗∗ p value < 0.0001. (B) We could validate 16 out of 20 tested Alu exons that show a more than two-fold increase in inclusion in the RNA-seq data of at least one of the HNRNPC knockdowns (Table S3). Validations were performed and presented as in (A). (C) Control RT-PCR experiments analyzing splicing changes of exons that are known to be regulated by TIA1/TIAL, PTB, hnRNP A1 and TDP-43 (see Table S3C). Experiments were performed in triplicates using the two HNRNPC knockdowns (KD1 and KD2), a TIA1/TIAL double knockdown, a PTB/nPTB double knockdown, individual knockdowns for HNRNPA1 and TDP-43 (all labeled as KD with the respective gene(s) indicated above) as well as control (Ctrl) HeLa cells. The gel view of capillary electrophoresis of the PCR products (right) and the signal quantification (bar diagram on the left) are shown for each exon. The sizes of the quantified fragments including (in) or excluding (ex) the regulated alternative exon are marked on the right of the gel image (Table S3). Bar diagrams on the right depict the me [file mmc5.pdf]

S1A (Part 1 of 3)

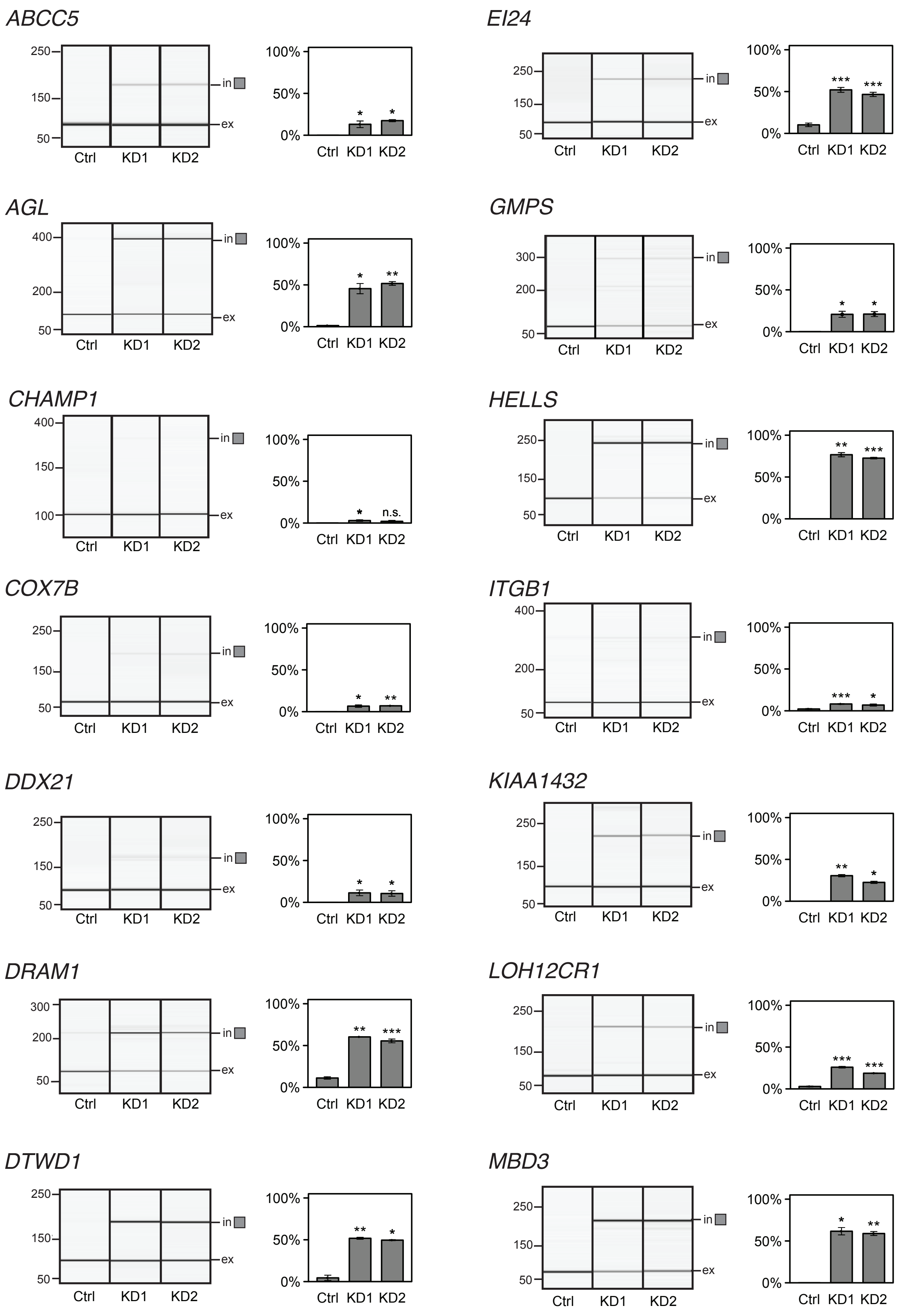

S1A (Part 2 of 3)

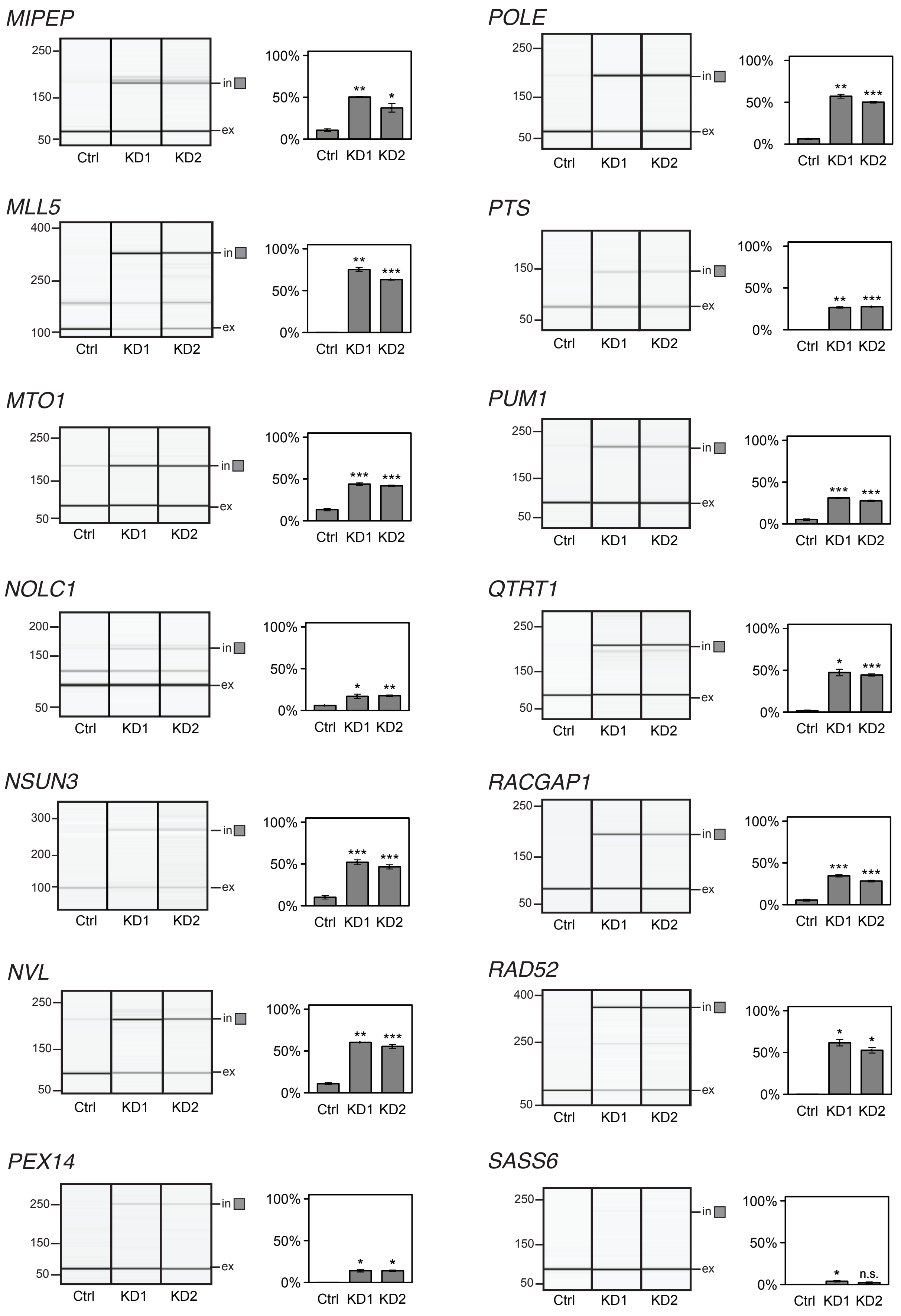

S1A (Part 3 of 3)

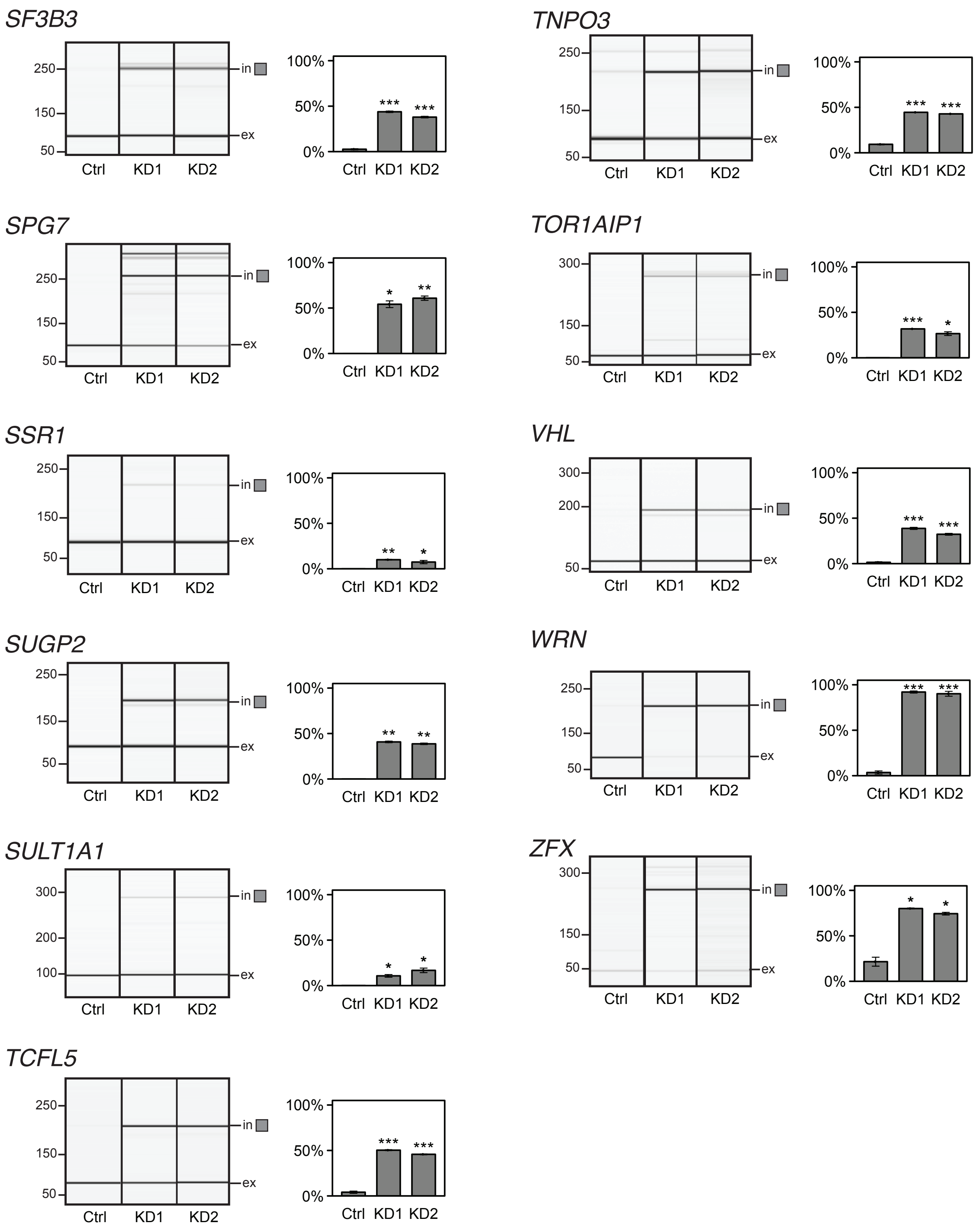

S1B

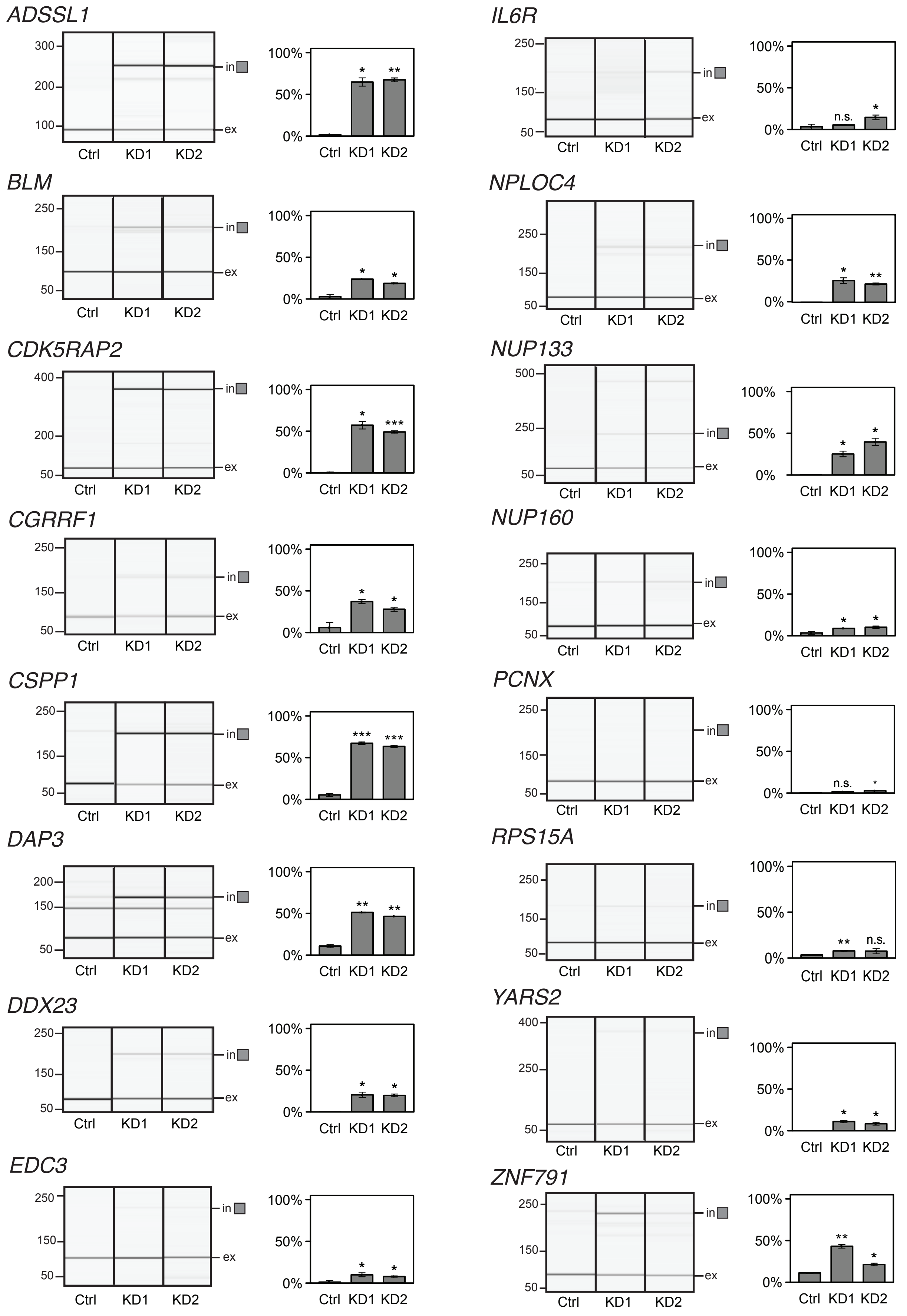
ADSSL1
300
200
100
in
ex
Ctrl KD1 KD2
100%
50%
0%
*
**
Ctrl KD1 KD2
IL6R
250
150
50
in
ex
Ctrl KD1 KD2
100%
50%
0%
n.s.
*
Ctrl KD1 KD2
BLM
250
150
50
in
ex
Ctrl KD1 KD2
100%
50%
0%
*
*
Ctrl KD1 KD2
NPLOC4
250
150
50
in
ex
Ctrl KD1 KD2
100%
50%
0%
*
**
Ctrl KD1 KD2
CDK5RAP2
400
200
50
in
ex
Ctrl KD1 KD2
100%
50%
0%
*
***
Ctrl KD1 KD2
NUP133
500
250
50
in
ex
Ctrl KD1 KD2
100%
50%
0%
*
*
Ctrl KD1 KD2
CGRRF1
250
150
50
in
ex
Ctrl KD1 KD2
100%
50%
0%
*
*
Ctrl KD1 KD2
NUP160
250
150
50
in
ex
Ctrl KD1 KD2
100%
50%
0%
*
*
Ctrl KD1 KD2
CSPP1
250
150
50
in
ex
Ctrl KD1 KD2
100%
50%
0%
***
***
Ctrl KD1 KD2
PCNX
250
150
50
in
ex
Ctrl KD1 KD2
100%
50%
0%
n.s.
*
Ctrl KD1 KD2
DAP3
200
150
50
in
ex
Ctrl KD1 KD2
100%
50%
0%
**
**
Ctrl KD1 KD2
RPS15A
250
150
50
in
ex
Ctrl KD1 KD2
100%
50%
0%
**
n.s.
Ctrl KD1 KD2
DDX23
250
150
50
in
ex
Ctrl KD1 KD2
100%
50%
0%
*
*
Ctrl KD1 KD2
YARS2
400
250
50
in
ex
Ctrl KD1 KD2
100%
50%
0%
*
*
Ctrl KD1 KD2
EDC3
250
150
50
in
ex
Ctrl KD1 KD2
100%
50%
0%
*
*
Ctrl KD1 KD2
ZNF791
250
150
50
in
ex
Ctrl KD1 KD2
100%
50%
0%
**
*
Ctrl KD1 KD2

S1C

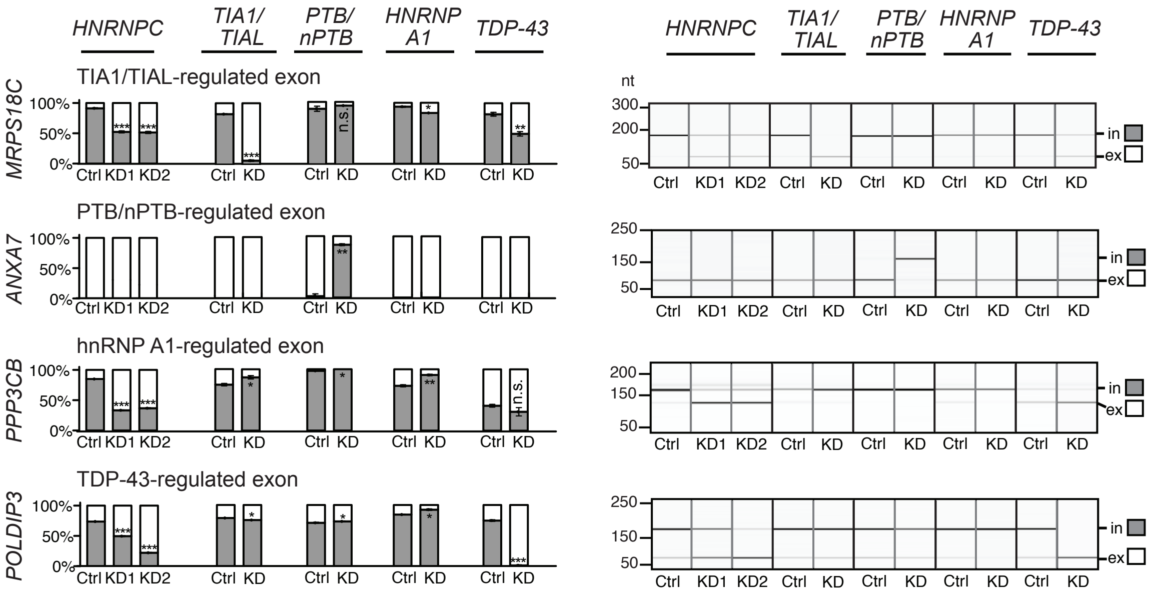

HNRNPC
TIA1/ TIAL
PTB/ nPTB
HNRNP A1
TDP-43
TIA1/TIAL-regulated exon
MRPS18C
PTB/nPTB-regulated exon
ANXA7
hnRNP A1-regulated exon
PPP3CB
TDP-43-regulated exon
POLDIP3
100%
50%
0%
Ctrl KD1 KD2 Ctrl KD Ctrl KD Ctrl KD Ctrl KD
n.s.
nt
in
ex
